# Skeleton Graph-Neural-Network-Based Human Action Recognition: A Survey

**DOI:** 10.3390/s22062091

**Published:** 2022-03-08

**Authors:** Miao Feng, Jean Meunier

**Affiliations:** Department of Computer Science and Operations Research, University of Montreal, Montreal, QC H3C 3J7, Canada; miao.feng@umontreal.ca

**Keywords:** skeleton graphs, human action recognition, graph neural networks, survey

## Abstract

Human action recognition has been applied in many fields, such as video surveillance and human computer interaction, where it helps to improve performance. Numerous reviews of the literature have been done, but rarely have these reviews concentrated on skeleton-graph-based approaches. Connecting the skeleton joints as in the physical appearance can naturally generate a graph. This paper provides an up-to-date review for readers on skeleton graph-neural-network-based human action recognition. After analyzing previous related studies, a new taxonomy for skeleton-GNN-based methods is proposed according to their designs, and their merits and demerits are analyzed. In addition, the datasets and codes are discussed. Finally, future research directions are suggested.

## 1. Introduction

Human action recognition (HAR), aiming at automatically detecting human activities, has become increasingly popular, especially after being armed with deep learning, tremendous data and more computational resources. Typically, HAR holds great value in video surveillance [1,2], human–computer interactions (HCI) [3,4,5], virtual reality [6,7,8], security [9] and so forth.

HAR is supported by multi-modalities. Specifically, one kind of modality is structured data, e.g., images or videos and auxiliary data, such as semantic information. The common use of sensors (including cameras) and cloud databases makes structured data easy to be captured and shared. Moreover, they are visually or semantically informative, e.g., the shape or motion difference of subjects, the space–time trajectory [10] and the names of joints.

With the help of carefully designed representation learners, such as deep-learning (DL) models, these informative representations are obtained in a task-related way so as to help solve the problem more accurately. However, the performances are upper-bounded by the data, which emphasizes less on the intrinsic relations between the joints of skeletons. The other is unstructured data that are non-Euclidean, such as human skeletons. Extractors, e.g., Openpose, Google PoseNet and Nuitrack, are capable of working in real-time and thus generate sufficient skeleton graphs.

These poses contain intrinsic information among spatial joints and temporal frames as well as 3D information if the depth data are offered. Additionally, compared with an image that requires a storage space proportional to the image width, height and number of channels, skeletons only require the 3D coordinates and confidence score of every joint, and normally there are no more than 30 joints, which decreases the storage cost significantly.

Moreover, while image-based methods suffer from varied brightness, changing of backgrounds, chromatic differences, different subjects etc, 3D skeletons can work on various scenes once they are detected. As HAR should label the same activity with the same label even when performed by different persons under different conditions or styles, a skeleton graph is undoubtedly a promising choice.

Models to find representations of human skeletons are classified into three categories.

**Figure 1:**
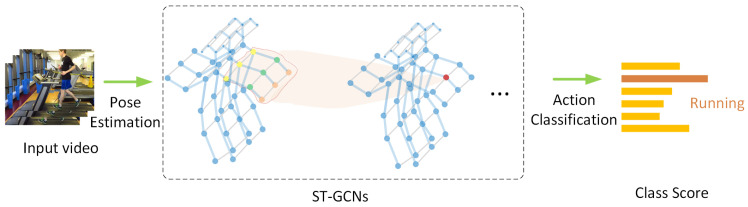
The method ST-GCN [13].

**Figure 2:**
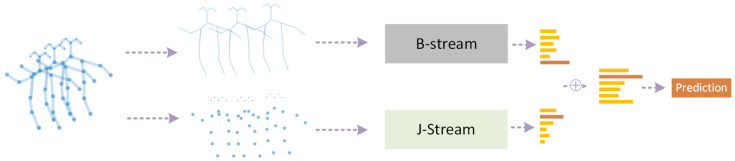
The method 2s-AGCN [14], B-Stream and J-Stream stand for bone stream and joint stream, respectively.

- The traditional method is handcrafted descriptors, such as principle components analysis (PCA) based on 3D position differences of joints [11], selecting joint pairs by top-*K* Relative Variance of Joint Relative Distance [12]. These descriptors are interpretable; however, they are limited as they tend to extract shallow and simple features and normally fail to find significant deep features.
- The other idea is redefining the problem a deep learning problem in Euclidean space, such as serializing the graph nodes into a sequence and then adopting the well-known Convolutional Neural Networks (CNN), Recurrent Neural Networks (RNN) etc. In this way, deep features are extracted mechanically but without paying attention to the intrinsic spatial and temporal relations between graph joints, e.g., the serialization of joints ignores their natural structures in skeletons.
- Recently, Graph Neural Networks (GNNs), especially graph convolution networks (GCNs), have come into spotlight, and were imported into skeleton graphs. The earliest milestone is ST-GCN [13] (Figure 1). Thereafter, multiple works based on ST-GCN were proposed. Among them, 2s-AGCN [14] (Figure 2) is another typical work, which adopted an attention mechanism. As GNNs are professional in discovering the intrinsic relations between joints, GNN HAR methods have achieved a new state-of-the-art (SOTA).

**Figure 3:**
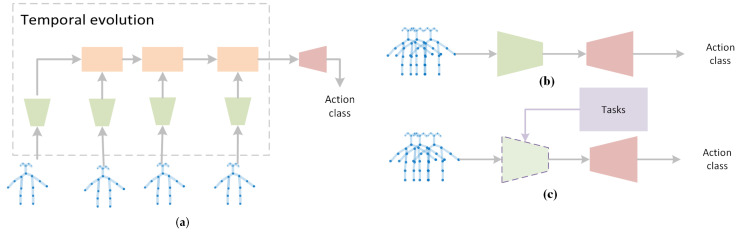
Illustration of (**a**) the spatial-based approach, (**b**) the spatiotemporal-based approach and (**c**) the generated approach. Among the figures, green trapezoid blocks denote the GNN-HAR model with dashed lines marking the unfixed model structure; pink trapezoid blocks denote action classifiers; orange trapezoid blocks for hidden states and purple blocks for tasks rather than HAR. The multiple skeletons together in (**b**,**c**) stand for skeleton graphs of video clips. The purple arrow in (**c**) denotes supervision from tasks, such as adversarial learning and knowledge distillation.

Therefore, this survey focuses on skeleton GNN HAR, which regards the input skeleton as a graph and process it via GNNs. It is true that there are many HAR surveys; however, most of them emphasize sensors, DL methods, datasets or HAR applications. The rapid development of GNNs and emergence of GNN HAR methods call for a constant update. As far as we know, there is only one survey that analyzed GCN-based methods; however, it did not cover all aspects and missed some approaches. In this respect, our survey updates and completes the aforementioned existing survey. The main contributions of this survey are as follows:

1. New taxonomy: We propose a new taxonomy for previous methods, which relate to GNNs and skeleton graphs. They are grouped into spatial-based approaches, spatiotemporal-based approaches and generated approaches. Figure 3 illustrates the idea. Their common frameworks are also summarized.
2. Comprehensive review: Apart from analyzing methods, we also review the categories of skeleton graphs in applications and the construction of them.
3. Abundant resources: To give a complete summary for the skeleton-GNN-based HAR, we collected commonly used datasets and published codes. The details of each collected dataset and method are summarized in Table A1 and Table A3, respectively, in Appendix A and Appendix B.
4. Future directions: Further directions are presented and discussed after having a look at the challenges in this field, with the hope of offering some inspiration for the benefit of other researchers.

**Figure 4:**
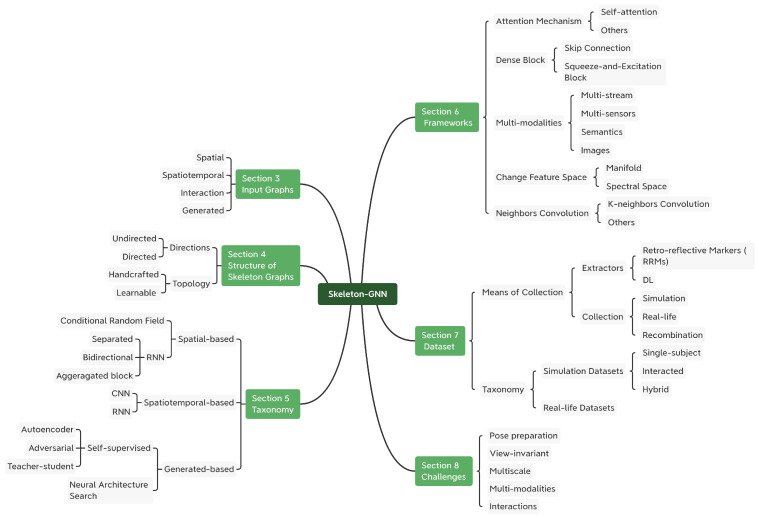
The framework of this paper, where RNN means recurrent neural networks, CNN means convolutional neural networks and DL means deep learning.

This paper is organized as follows: Section 2 gives a literature review on GNNs and HAR. Then, Section 3 analyzes skeleton graphs more specifically, leading to Section 4, which focuses on how to build skeleton graphs. Afterwards, the new taxonomy of methods on skeleton GNN HAR is proposed in Section 5, and their common frameworks are described in Section 6, which gives a comprehensive review for all related approaches. Various datasets are collected in Section 7, and then we end with a discussion of the challenges and our final conclusions in Section 8 and Section 9, respectively. The Appendix A and Appendix B collect all mentioned datasets and methods. A detailed framework of this survey is presented in Figure 4.

## 2. Previous Works

### 2.1. GNNs

Recently, there has been an increased interest in graph data. Their applications include e-commence recommended systems, chemistry molecules, citation networks and so on. Some works are committed to extract embeddings, either at node-level, edge-level or graph-level embeddings. Some are more interested in topologies attempting to add edges or nodes to build a new topology or regenerate a graph after observing subgraphs [15].

Nodes in a graph do not satisfy the independent and identical distribution (i.i.d.) assumption [15]. In contrast, each node is related to others through various types of links. When this dependency presents troubles, it also contributes to the intrinsic information.

Motivated by the requirement of mining graph data, the name of GNNs was first introduced by Gori et al. [16] in 2005. Afterwards, by extending the achievements of CNNs and RNNs, convolutional graph neural networks (ConvGNNs) and recurrent graph neural networks (RecGNNs) were improved gradually. For ConvGNNs, both the spectral ConvGNNs and spatial ConvGNNs were developed. Spectral ConvGNNs prefer graph kernels in spectral space, while spatial ConvGNNs imitate traditional CNNs but perform on the *k*-order topological neighbors. Apart from them, many alternatives have emerged, including graph autoencoders (GAEs) and spatiotemporal graph neural networks (STGNNs).

Considering the accomplishment of GNNs, if the skeletons are built as graphs, then any GNN proposal can be a possible candidate for skeleton graph-based HAR.

### 2.2. HAR Surveys

Surveys of HAR have been studied by many researchers. Since 2010, according to the topics, related papers are mainly classified as:

1. Papers on datasets [17,18], which reviewed the common used datasets in HAR tasks.
2. Papers on modalities [10,19,20,21,22,23] on videos (images) [24,25,26] on skeletons, and [27] analyzed multi-modalities.
3. Papers on sensors, among which, paper [28] is related to inertial sensors [29] analyzed kinect-related approaches [30] on depth sensors, and [31] on body-worn sensors.
4. Papers on methodologies, where [32] dived into GCN-based approaches [23,33,34,35,36,37] collected DL-based methods, and [38] collected both handcrafted-based methods and learning-based methods. Specifically [36,37] only summarized CNN-based approaches, while others analyzed all kinds of DL approaches.
5. Papers on evaluation, such as [39], gathered the evaluation metrics applied on HAR tasks.
6. Papers on applications. Surveys, such as [40] on human–robot interaction applications, [41] on automatic driving, [42,43] on view-invariant domain, [44] on multi-people video surveillance, [45] on gestures, and [46] in the traffic context.

Although these surveys attempted to review the new emergence of HAR, only Tasweer et al. [32] focused on GCN-based approaches. Papers [26,29] mention GCN-based methods but do not take them as their main purpose.

Tasweer et al. set their point on GCNs, and proposed a taxonomy that categorizes GCN-based HAR into five architectures, which are spatiotemporal GCN, RNN-Attention GCN, Two-Multi Stream GCN, Encoder–decoder GCN and Miscellaneous GCN. They are the first to discuss such taxonomy for GCN-based HAR; however, they emphasized this taxonomy without analyzing other aspects, such as the types of graphs, the construction of graphs, and thus it is not a thorough survey. Moreover, they mix the category of GCN methods and generalized frameworks together, e.g., the ‘Two-multi Stream’ is a common framework, while ‘RNN-Attention GCN’ is a method with specific networks. Their taxonomy is kind of confusing when one attempts to understand a proposed method systematically. This motivated our paper where a deeper analysis of skeleton-GNN-based HAR is offered.

Specifically, rather than mixing GCN categories and frameworks together, we summarize the ways to construct skeleton graphs, the category of used networks, and the frameworks that can be generalized, into four sections. Section 3 and Section 4 discussed the categories and structures of skeleton graphs, Section 5 collects the main approaches, and Section 6 demonstrates the common frameworks. For graphs, this paper emphasizes the categories of skeleton graphs in applications and their structures and also introduces the ways to build them.

For approaches based on whether to create an end-to-end model and the input skeletons, this paper classifies skeleton-GNN-based HAR methods into spatial approaches, spatiotemporal approaches and generated approaches. Among them, the spatial approaches and spatiotemporal approaches use static models and are trained in end-to-end mode. However, for generated approaches, models are first trained in a non-end-to-end way. A non-task-specific model is trained before, and the task-specific model is obtained based on this pretrained model.

## 3. Skeleton Graphs in Applications

### 3.1. Input Graphs

Although the most direct idea is extracting a graph for each frame, the used graphs vary in real applications.

#### 3.1.1. Spatial Graphs

The simplest idea is taking the skeleton from each frame as an independent graph. In this way, the graph size will be the number of skeleton joints, and the graph links are the physical connections. If representing the graph as $G=\;\left(V,E\right)$, where the node set V is a set consisting of body joints, featured by 3D/2D coordinates and confidence scores; and the edge set E contains the links of physical connections.

Usually, edges can be represented as an adjacency matrix A for further utilization, with each item denoting whether there is a link between two nodes. Concretely, $A_{ij}=1$ denotes there is a link between joint *i* and joint *j*, and $A_{ij}=0$ for null edges.

#### 3.1.2. Spatiotemporal Graph

**Figure 5:**
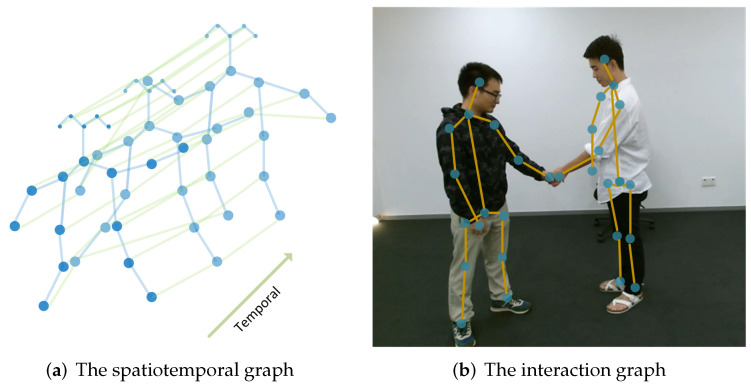
The input graphs, (**a**) with the green color to denote the temporal dimension and blue to denote the spatial dimension; (**b**) demonstrates shaking hands [47].

The other idea is extending the spatial graph by connecting skeleton joints along temporal dimension to build a spatiotemporal graph, e.g, the graph used in ST-GCN. In this way, both the spatial and temporal information are combined to use. However, taking the meaning of edges into consideration, this graph is heterogeneous since one type of edges coming from physical connections and the others explicitly display temporal relations. Figure 5a demonstrates the idea.

#### 3.1.3. Interaction Graphs

When performing GNNs on human-interaction applications, a graph that consists of two or more skeletons was proposed [48], one example is shown in Figure 5b. In this way, nodes are heterogeneous since they come from different subjects, and edges are also heterogeneous because one type of edges are the physical connections within each skeleton, and the other type is the connections between subjects.

#### 3.1.4. Generated Graphs

**Figure 6:**
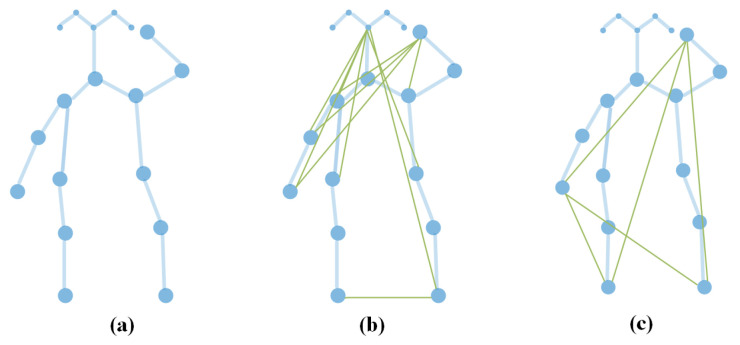
The generated graph [49], where (**a**) the original skeleton for the action phone call and (**b**,**c**) illustrates the inferred action-specific skeletons for the action phone call with new edges in green.

The graphs mentioned before were all manually built; however, in some applications [49], the authors assumed that the graph was redundant or uncompleted, which is frequent if there are occlusions, and thus they attempted to generate a new graph. In this way, the graph is modified with automatically generated nodes or edges, which are beyond the idea of spatial, temporal or interaction links. Figure 6 demonstrates an example where action specific edges are added.

### 3.2. Problem Definition

The problems to be solved for different input graphs vary. In the spatial graph, the main point is how to extract spatial information and then perform the temporal aggregation to obtain long-term information. For the spatiotemporal graph, the essential difficulty is to process the spatial and temporal information simultaneously. In the interaction graph, another problem is how to preserve interactions. As for the generated graph, adding the relevant information is the main challenge.

Moreover, when the graph is built as a directed graph (see next section), the main problem is how to pass messages efficiently. Since, in this graph, messages can only pass along the predefined directions; therefore, the graph convolution kernels working on undirected graphs have to be modified to fit. Based on the forms of graphs used in HAR, the next section will take a deep look at the structure of skeleton graphs.

## 4. The Structure of Skeleton Graphs

### 4.1. Graph Structures Based on Directions

Before extracting the spatial and temporal information, the essential step is to build a graph. Based on whether the input graphs are directed or undirected, usually, graphs are further combined with GNNs after converting edges as a connection matrix, such as an adjacency matrix. For directed graphs, connection matrices are more complex since they have to denote the predefined directions to pass messages accordingly.

#### 4.1.1. Undirected Graphs

Most methods are built upon undirected graphs. ST-GCN is one well-known milestone. Most Graph Neural Networks (GNNs) are first proposed for undirected graphs, among which, messages are conducted in bi-directions. The adjacency matrix in this case is symmetrical and therefore leads to even-handed passing of information on each orientation. One clear demerit of the undirected graph is that it does not cover direction information. For example, bone information, which represents the direction and length of a bone, has been proven to be a good modality for skeleton graph-based HAR [16,50].

#### 4.1.2. Directed Graphs

Methods built on directed graphs are rare, e.g., [50,51,52]. Though the computations are more expensive in these graphs since not each connection direction is equal, they are capable of emphasizing the messages for action related parts, such as arms for clapping and legs for jumping.

Lei Shi et al. [50] and B. Fu et al. [51] handled joints and bones information simultaneously and thus proposed to take joints as nodes and bones as edges. The center of gravity in the skeleton is defined as the root node, and the direction of each edge (bone) is determined by the distance between the node and the root node. The node closer to the root node is designated to point to the node farther from the root node.

Q. Zuo et al. [52] built a directed graph for each body part, more precisely, left arm, right arm, left leg, right leg and trunk. The connection matrices used in their directed graph consist of a self-loop matrix (an identity matrix), an inward and an outward adjacency matrix. The inward and outward connections are determined with respect to the center point in that part. Each connection matrix is tackled by one GCN layer, and finally features from these three matrices are fused together as the part features.

J.L. Gao et al. [53] regarded an undirected spatial temporal graph as two directed graphs with opposite directions for each edge, and therefore the message passing between joints is bi-directional, leading to two directed graphs, namely the focus graph and diffusion graph. During message passing, they first convey information forwardly through focus graphs and then transform the updated features back via diffusion graphs.

### 4.2. The Construction of Graph Topology

The construction of graph topology is essential since topology holds a great value of structural information. Here, the topology not only covers the edges in the graph but also the nodes. In this respect, the frame-level, edge-level or node-level will be discussed. The construction absorbs ideas from handcrafted topology and learnable topology, while handcrafted topology is simple and cheap in computation, learnable topology is more adaptive as it attempts to build a new graph to preserve relevant information.

#### 4.2.1. Handcrafted Graph Topology

##### Modality Level

Rather than directly take the skeletons as a graph, some papers created the skeleton graph from implicit features to complete the information that original skeleton graphs missed. For example, J. Cai et al. [54] took the joint-aligned optical flow patch sequence as an orthogonal cue to the skeleton sequence, and then tackled features from this sequence as an implicit graph.

##### Frame Level

In this level, the easiest way is downsampling the frames, such as what Z. Liu et al. [55] proposed. They chose a frame every *d* frames and named this operation as dilated windows with the aim to enforce the information across spatiotemporal dimension but decrease the redundant information aggregated from an increasingly larger spatiotemporal receptive field.

##### Subgraph Level

Some researchers are more interested in subgraphs—in other words, sub-connection matrices, e.g., divided or factorized adjacency matrices. Since, after division, different matrices can be flexibly manipulated with different weights, this provides the ability of paying diverse attention on different parts. This idea also helps to capture local information.

- Body-part partition
  Papers, such as [56,57,58,59,60,61,62], directly divided the original skeleton graph into several body parts. Typically, the group comprising left arm, right arm, left leg, right leg and trunk, is intuitive and easy to be implemented. Normally, our limbs are more flexible than our trunk and interact more with other parts. Moreover, when people are moving, the diverse parts of the human bodies are capable of making distinct gestures. Based on this, many strategies can be designed to assign different weights strategy to these parts.
- Distance-based partition
  In this category, the definition of distance mainly focuses on centrifugal and centripetal partition, which divides the neighbors of each node into two or more parts. For one node $v_{i}$, a node $v_{j}$ in the centripetal part is closer to the gravity center than $v_{i}$, and a node $v_{k}$ in the centrifugal part is farther away from the gravity center than $v_{i}$. Usually, the gravity center is the average of all skeleton joints. Although this idea does not partition the graph topology explicitly, the adjacency matrix is implicitly classified into different groups with an allowance of applying different weights.
  The idea was first proposed by ST-GCN that divides node $v_{i}$’s neighbors into the group $v_{i}$, the group $v_{i}$’s centripetal joints and the group $v_{i}$’s centrifugal joints. The other case comes from [63], which suggests making use of neighbor bones of node $v_{i}$ and therefore augmenting the three partitions to five, with the addition of centripetal bones and centrifugal bones, where the centripetal bones are those that are closer to the gravity center than $v_{i}$, and the centrifugal bones are those that are farther from the gravity center than $v_{i}$.
- Multiscale partition
  One partition is based on geometry. For example, B. Parsa et al. [64] performed GNN on node-level, part-level and global-level graphs. The global level graph is the output of the group average pooling on the part-level graph, and the part-level graph is the output of group average pooling on the node-level graph (the original skeleton graph).
  The other partition directly makes use of downsampling so as to implement it mechanically. For example, Y. Fan et al. [65] conducted two more downsampling operations to extract additional graphs of different scales from the original graph.

##### Edge Level

Apart from the common used self-loop matrix and adjacency matrix, some researchers argue that there are more implicit edges. Some emphasize the edges in temporal dimension. O. Yuya et al. [66] found new edges by a proposed temporal extension module, which adds connections to multiple adjacent joints on inter-frames. To expand the sampling area for the temporal dimension gradually, the proposed temporal extension module is applied between conventional spatial graph and temporal graph convolution.

P. Ghosh et al. [67] added more temporal connections that can span multiple timesteps, e.g., the left arm joint at timestep *t* can have connections with corresponding joint at timestep t+1,t+2,⋯ rather than only t+1 in ST-GCN. To capture the dependence of non-physical connections between joints, some researchers simply add edges between joints, while others focus on edges between the parts of interest.

Z. Bai et al. [68] fully connected each joint in the skeleton with other joints arguing the division of root, centripetal and centrifugal in ST-GCN is not optimal.

R. Zhao et al. [69] added edges between limbs and head, while treating all other joints independently and named this graph as the global graph. The original skeleton graph was taken as the local graph. Finally, they computed the sum of the output from both local and global graphs.

Y. Li et al. [70] focused on hand gesture recognition. They added three types of edges, one by linking the tip of each finger except the pinkie with the base of the finger to its right, and the tip of the pinkie is linked with the base of the ring finger. The second type is to link the third joint of each finger except the pinkie to the second joint of the little finger. The third type is to link the tip and third joint of the same finger. Among them, the first type offers a way to measure the distance of two adjacent fingers, the second better measures the opening degree of the hand. The third type directly provides the information of some actions, such as grabbing in which fingers bend.

Moreover, some methods inherit the characteristics of actions and physical nature, such as symmetry and movements. Q. Zuo et al. [52] added a symmetric matrix and edge matrix, among which, the symmetric matrix considers the symmetric structure of the human body, and the edge matrix tends to contain significant movements of some edge joints. The inspiration comes from the fact that, if one waves his hands, then the movement of hands is more clear than the arms, considering the acceleration.

Additionally, some methods choose a constant hyperparameter to generate edges. In addition to the self-loop and common adjacency matrix, X. Hao et al. [71] added global hyperedges capturing global information and local hyperedges capturing local information. These edges are generated by varying the regularization parameter of sparse representation objective functions. In other words, global and local hyperedges are constructed under different sparsity assumptions, controlled by a hyper parameter β. The choice of β is in a specified range.

#### 4.2.2. Learnable Graph Topology

Learning graph topology is more adaptive, which allows finding more valuable information and keeping the information of interest.

##### Frame-Level

At the frame-level, except for manually selection, an automatic mechanism armed with reinforcement learning (RL) is preferred. To build a RL system, three components—namely *rewards*, *values* and the set of *actions*—are unavoidable. For each *action*, such as choosing one frame or dropping one frame, a predefined *reward* will be assigned. After evolving for a sequence of *actions*, *values* are used to estimate the current state. The objective of a RL system can be defined to obtain a highest *value* at the preferred *state*. After carefully designing the *reward* mechanism, RL is capable of choosing key frames and ignoring frames with unclear motions or other irrelevant information.

Y. Tang et al. [72] proposed the Progressive Reinforcement to detect the best key frame, the set of *actions* consists of a shift to the left, shift to the right and staying still. The *state* is at the chosen frame at time *t*, and *rewards* are $\left[1,-1\right]$ at the first iteration for correctly predicting or not; after that, the *rewards* are [Ω,−Ω], where Ω is a constant. Deep Q-learning and policy gradient are adopted in two branches. Frame distillation network (FDNet) and GCN promote each other mutually, as GCN provides *rewards* for FDNet and FDNet selects key frames to refine GCN. The better the GCN is, the more accurate *rewards* will be provided.

##### Edge-Level

To capture more information among physically nonadjacent joint but action-related or task-related joints, most methods focus on learning new connections. Some authors formalize the learning procedure as a learnable matrix by modifying existing methods. L. Shi et al. [73] adopted two new parameterized adjacency matrices rather than the original adjacency matrix, namely $B_{k},C_{k}$, where *k* denotes the index of layer in the model.

Moreover, $B_{k}$, the global graph learned from data, represents the graph topology that is more suitable for the action recognition task; $C_{k}$ is the individual graph learned by normalized embedded Gaussian function, which has unique topology for each sample.

H.Y. Yang et al. [74] proposed a learnable matrix that can learn pseudo connections, covering the dependencies between connected joints and joints that are not connected. Other researchers propose an explicit inference module to generate a new adjacency matrix.

- For every three consecutive frames, X. Gao et al. [75] proposed learning a new graph with a graph regression (GR) module. The graph regression problem is formulated as the optimization of the graph Laplacian matrix L. For intra-joints, the weights for weakly connected and strongly connected joints are different, where strong connections include physical connections and some physical disconnections among joints, and weak connections denote potential connections, such as those between head and hands. For the inter-joints, connections between corresponding joints along the temporal dimension and their neighborhoods are assigned with different weights, and others are set as zero.
- To capture the intrinsic high-order correlations among joints, B. Li et al. [76] proposed spatiotemporal graph routing, consisting of a spatial graph router (SGR) and temporal graph router (TGR). SGR captures the connectivity relationships among joints based on sub-group clustering. TGR focuses on structural information with the correlation degrees of joints trajectories.
- M. Li et al. [77] estimated actional links (A-links) and structural links (S-links), where A-links are estimated by encoder–decoder (AE)-based A-links inference module (AIM), and S-links are estimated by high-order polynomials of an adjacency matrix. The A-links capture the latent dependencies among joints, and S-links indicate higher order relationships.
- F. Ye et al. [78] proposed a joint relation inference network (JRIN) to aggregate the spatiotemporal features of every two joints globally and then infer the optimal relation between every two joints automatically. The relations of joints are quantified as the optimal adjacency matrices.
- F.F. Ye et al. [79] estimated edges by joint-relation-reasoning (JRR). JRR is trained by RL, optimized with policy gradient. In detail, the *state* equals to E⊗M, where E contains the global edges information, M represents the connectivity weights of every tow joints, and ⊗ denotes the element-wise product; *rewards* comes from the output of GCN; *action* is the output of JRR, which indicates temporal relevance of every two joints under the current *action*.
- To extract the implicit connections and properly balance them for each action, W.S. Chan et al. [49] created three inference modules, namely the ratio inference, implicit edges inference and bias inference. The finally estimated matrix is the combination of the output from these three modules. The ratio of the implicit and structural edges is vital. Adjacency matrices A and $M_{bias}$ present the structural edges. $M_{bias}$ is updated with back propagation and is kept the same for all actions.

Other works show their interest in context-enriched skeleton, samples variety etc. Fanfan Ye et al. [80] proposed learning a context-enriched dynamic skeleton topology with a Context encoding Network (CeN). CeN simply consists of three 1×1 convolutional layers and permutations, which maps the input tensors into an adjacency matrix. The convolution is alongside the joint coordinate dimension, temporal dimension and then joint dimension, and thus CeN can generate sample different graphs.

K. Liu et al. [63] proposed learning additional connections among joints and bones for various action samples. Precisely, the adaptive joint-bone adjacency matrix and adaptive joint adjacency matrix are all learned by softmax, which uses normalized embedded Gaussian functions to measure similarity.

##### Node-Level

Rather than focusing on new connections, some methods aim to remove redundant information or aggregate messages from multiple nodes. Some works learned new vertexes—in other words, they aggregate multiple nodes as one node. Heidiri et al. [81] proposed a spatiotemporal bilinear network (ST-BLN) with no requirement of predefined adjacency matrix. ST-BLN forces the attention matrix to be symmetric. The selection of the nodes in the first layer will lead to an aggregation of joint information or the addition of new nodes.

Other works selected nodes first. To distinguish the most informative joints for each stream, Y.F. Song et al. [82] only passed the information from unactivated joints to the next stream. The activated class activation maps (CAM) obtained from previous GCN streams are accumulated as a matrix to inform the new stream about which joints have been already activated. Others aim at learning node embeddings.

- W.W. Ding et al. [83] emphasized the learning of localized correlated features. By projecting each part of human body into a node, a fully connected similarity graph is formed to capture relations among the disjoint and distant joints of the human body. The learned mapping of spatial matrices and temporal matrices can determine which part of the human body across several consecutive frames should be mapped to a node in the similarity graph.
- W.J. Yang et al. [84] merged nodes in the same part of the skeleton into one node. Each new generated node takes the weighted summation of the original nodes that it covers as its feature, using trainable weights. This integration is done part-wise and channel-wise.
- Y.X. Chen et al. [85] proposed structural pooling since the motion information contained in human body is highly related with the interaction of five body parts, and therefore graph convolution on the graph with these five-part nodes can capture more global motion information. By graph pooling, the new compressed graphs in different sizes are input to the model.
- G.M. Zhang et al. [86] proposed learning a new topology by topology-learnable graph convolution, which is decomposed as feature learning and node fusion. Node fusion is performed by a learnable fusion matrix L that is initialized with a normalized adjacency matrix and added with an additional constant bias.

## 5. A New Taxonomy for Skeleton-GNN-Based HAR

The skeleton-GNN-based HAR approaches are classified as spatial methods, spatiotemporal methods and generated methods, while spatial ones take spatial graph as input, spatiotemporal ones use spatiotemporal graphs as inputs, and generated approaches are supervised by tasks rather than HAR, such as knowledge distillation, or have an unfixed model structure before training. The idea is illustrated in Figure 3.

### 5.1. Spatial-Based Approaches

**Figure 7:**
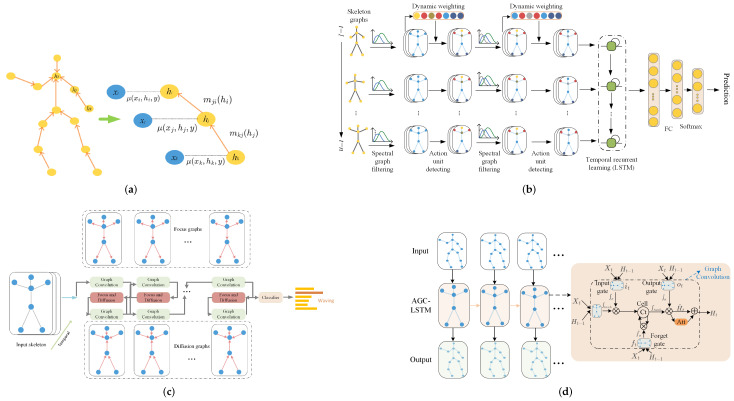
Examples of spatial methods: (**a**) the CRF approach [87], the others are RNN methods. Precisely, (**b**) a separated approach [88], (**c**) a bidirectional approach [53] and (**d**) the aggregated approach [89].

Approaches in this category take GNN as a spatial feature extractor, and the temporal evolution is handled by other modules. Two major candidates are proposed to evolve states in temporal dimension. One category is traditional conditional random field (CRF) methods, including Hidden CRF (HCRF). The other one prefers the family of RNN, such as RNN, long-short temporal memory network (LSTM) and Gated Recurrent Units (GRU). Examples in each category are shown in Figure 7.

#### 5.1.1. CRF

CRF is an undirectional graph model whose nodes are divided into exactly two disjoint sets X and Y, the observed and output variables, respectively. The conditional distribution $p\left(Y|X\right)$ is then modeled. It is suitable for labeling action sequences since Markov chain models are able to track the evolution among temporal dimension.

K. Liu et al. [87,90] argued that GCN is powerful in extracting spatial information but weak on state evolution and then performed HCRF on extracted features. After obtaining features by GCN, HCRF will learn hidden states on each node and perform directed message passing on these hidden states. Finally, under the minimum negative conditional log-likelihood rule, the label for an action sequence sample is defined. By viewing the skeleton graph as a CRF, K. Liu et al. [63] adopted CRF as a loss function to improve performance.

#### 5.1.2. RNN

Although CRF works as a graph model and handles the state evolution, there are situations when they are non-Markov chains. For example, the current state may rely on states from all previous timesteps. This is why RNN was proposed and started becoming popular. The family of RNN is capable of preserving the relationships between states in multiple timesteps compared with CRF in *k* predefined timesteps. Among the family, LSTM is capable of solving gradient explosion and gradient vanishing that exists in vanilla RNN, while GRU can be regarded as a simplification of LSTM.

The RNN methods are classified as separated strategy, bidirectional strategy and aggregated block.

##### Separated Strategy

Some methods perform spatial information extraction, usually by GCN (either GCN in spectral space or in spatial space) and perform state evolution separately. In [88], to further encode continuous motion variations, the deep features learned from skeleton graphs by GCN in spectral space were gathered along consecutive temporal slices and then are fed into a recurrent gated network. Finally, the recurrent temporal encoding was integrated with the spectral graph filtering and action-attending to jointly train.

R. Zhao et al. [69] performed GCN and LSTM separately, the spatial information from GCN in each frame was directly input into LSTM cell. Z. Y. Xu et al. [91] proposed using RL combined with LSTM as the feature selection network (FSN) consisting of a policy network and a value network. To be precise, both the policy network and value network are based on LSTM for sequential *action* or *value* generation. The feature selection is done along temporal dimension and the input features are the spatial features from GCN.

S. Xu et al. [92] worked on two-subjects interaction graphs. After performing GNN on skeleton graphs in one frame to extract spatial information, the attentioned LSTM is preformed on the joint-level, person-level and scene-level so as to pass information in different scales. To leverage these three types of features, a Concurrent-LSTM (Co-LSTM) is applied to further balance their temporal dynamics for action recognition.

M.S. Li et al. [77] used GRU to update the joint features while inferring the future pose conditioned on the A-links and previous actions. The prediction from GRU evolution was then handled and later adopted by GNN.

In the work proposed by J.M. Yu et al. [93], RNN was used as an autoregressive model to predict the hidden state of noisy skeleton graphs. The hidden state was later used to predict action class. Q.Q. Huang et al. [94] worked with the same idea except for changing the basic GNN to attentioned GNN. Others, such as [62,64,95] extract state evolution information similarly after various GNN modules but not based on attentioned GCN.

##### Bidirectional Strategy

Considering the bi-directional information of video sequence, some use bidirectional LSTM to keep forward information and backward information simultaneously.

In order to utilize the past and future temporal information, X.L. Ding et al. [96] choose the bidirectional RNN to model skeleton sequences and adopt it before extracting spatial information by GNN. To capture the temporal contextual information over frames, J.L. Gao et al. [53] provide a context-aware module consisting of bidirectional LSTM cells, aiming at modeling temporal dynamics and dependencies based on the learned spatial latent nodes.

Except for the basic bidirectional LSTM, J. Huang et al. [97] deployed GCN on LSTM to enhance its ability of extracting spatial features. Precisely, they provided a LSGM that consists of one original LSTM cell followed by two GCN layers. Then, the LSGM was used to build Bi-Direction LSGM modules, which comprises of a forward LSGM and a reverse LSGM. The forward LSGM and reverse LSGM work in parallel, and the outputs from them are added together to pass to the next layer.

##### Aggregated Block

Some argue that the extraction of spatial information and temporal information can be stacked together as a basic building block; however, they process the spatial information before performing temporal convolution. Papers [89,98] integrated GCN with LSTM, in other words, each gate in LSTM—namely, the input gate, forget gate and output gate—is armed with GCN so as to operate LSTM directly on the extracted spatial information from each frame.

### 5.2. Spatiotemporal Approaches

**Figure 8:**
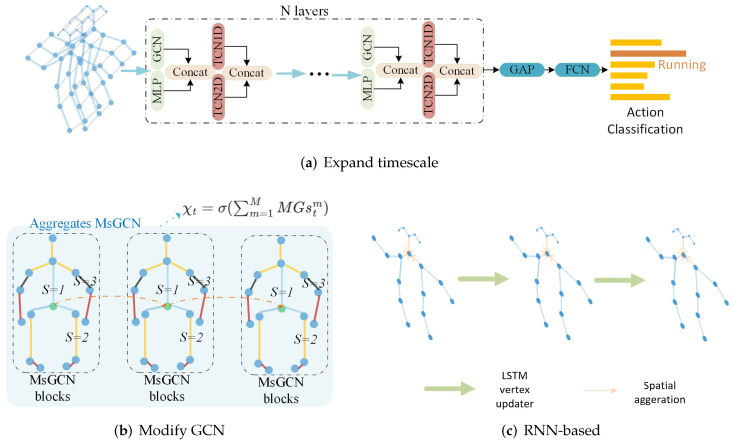
Examples of spatiotemporal-based methods: (**a**) the approach that attempts to expand timescale [99], (**b**) an approach that modifies GCN [100] and (**c**) an RNN-based approach [83].

The methods mentioned above tackle spatial information and temporal information separately. However, spatial information and temporal information are correlated. For example, the similar actions of Waking up and lying on the bed have similar spatial information but distributed at different timestamps. Examples in each category are shown in Figure 8.

#### 5.2.1. CNN

ST-GCN is a typical spatiotemporal approach since it performs GCN on spatiotemporal graph (STG) directly and therefore extracts spatiotemporal information simultaneously. Methods, such as [29,48,54,60,68,82,86,96,101,102,103,104,105,106,107,108,109,110,111,112,113] are all developed based on ST-GCN. Methods based on AGCN also work on STG, such as [66,73,93,114]. However, one drawback for these methods is that they only perform spatiotemporal extraction on a predefined temporal size (the kernel size of CNN in temporal dimension); therefore, multi-scale temporal information cannot be handled.

To work on multiple timescale dynamically so as to take either long term dependencies or short term dependencies into consideration, P. Ghosh et al. [67] also used STG but they allowed flexible temporal connections, which can span multiple timesteps. For example, the joint left arm at timestep *t* can have connections with left arm joint at timestep $\left(t+1,t+2,\cdots \right)$ rather than only at t+1 in ST-GCN. Their method is based on Hourglass (a CNN framework), combined with ST-GCN.

Z.T. Zhang et al. [99] attempted to handle temporal information with two gated temporal convolutional network (TCN), herein 1DCNN and 2DCNN with tanh and sigmoid activation functions working as gates. They argued that TCN will not overfit to some extent since it inherits the stable gradient of CNN. After performing filtering in temporal dimension, the outputs are combined together and then tackled by GCN and MLP.

In addition to making progress on temporal dimensions, some approaches attempted to modify GNN to take multi-scale in spatiotemporal dimension into consideration. Z. Hu et al. [100] established dependence relationships for different bone nodes with a bone joint module, which is based on multiscale dynamic aggregated GCNs. GCNs describe and aggregate the bone joint semantic information. In this way, either the spatial information or the multiscale temporal information are all handled together.

#### 5.2.2. RNN

Based on GCN, to tackle long-term information, W.W. Ding et al. [83] used LSTM as a vertex updater during message passing. Therefore, the features of each vertex will contain the temporal information and thus handle spatiotemporal information simultaneously.

### 5.3. Generated Approaches

The generated approaches cover two categories, one includes self-supervised methods, also known as unsupervised methods, and the other is neural architecture search (NAS), which aims at generating the best model by combining candidate components.

Both categories work in a non-end-to-end way. For the self-supervised methods, they first use priors, like pretext tasks, to generate a pretrained model, and then adapt it to fit the target task. For NAS, it aims at generating a best model on the target task. They emphasize the combinations of given components first, and chose the best model from these combinations. Then, the chosen model will be fine tuned on the target task.

**Figure 9:**
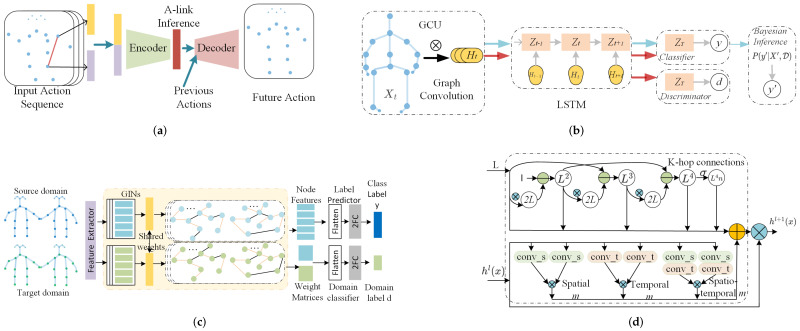
Examples of generated methods: (**a**–**c**) self-supervised approaches, where (**a**) the approach with Autoencoder (AE) [77], (**b**) the adversarial approach [69], (**c**) a teacher–student approach [115] and (**d**) a neural architecture search (NAS) approach [116].

Examples in each category are shown in Figure 9.

#### 5.3.1. Self-Supervised

Self-supervised learning is a means for training computers without manually labeled data. It is a member of unsupervised learning methods where outputs or goals are derived by machines. The machines are thus capable of labeling, categorizing and analyzing information on their own and then drawing conclusions based on connections and correlations. We classify methods in this category as AE, adversarial learning and teacher–student mechanism.

##### AE

M. Li et al. [77] built an A-links inference module (AIM) based on AE, where the output of the encoder is the probability of each joint pair with type-c link, and the decoder requires the output of encoder and joints positions in the previous frame. Thus, the loss of AIM is the difference between part of the input from the encoder and decoder’s prediction. In this way, no more labeled data are required during pre-training the AIM except for the input poses.

##### Adversarial Learning

Inspired by adversarial learning, in [69], they incorporated it into the Bayesian inference framework and formulated it as a prior that targets regularized model parameters as to improve the generalization. The discriminator was implemented as a fully connected layer. The loss function while training is similar as what is adopted in generative adversarial network (GAN).

##### Teacher–Student Mechanism

For transferring knowledge between two graphs, such as one obtained in the lab and the other from real life, Y.S. Tang et al. [115] used a teacher–student mechanism. The teacher network guides the student network to transfer the knowledge across the weight matrices by a task-specific loss function, so that the relation information is well preserved during transfer. By doing so, no more action labels for the target domain are required during training.

#### 5.3.2. NAS

In addition to self-supervised methods to generate task-specific models, some researchers showed their interest on automatic machine learning (AutoML), among which, NAS has gained more attention.

W. Peng et al. [116] discussed the best architecture of skeleton GCN methods, given components: the dynamic graph modules with various spatiotemporal cues and Chebyshev approximations in different orders. All candidates have residual connections. The proposed NAS framework works to find the most accurate and efficient network. Moreover, instead of providing a pre-defined graph, they generate dynamic graphs based on the node correlations captured by different function modules.

N. Heidari et al. [117] progressively adjusted the model topology by increasing the width of the model layers until the performance converges. If the addition of the last layer does not improve the performance, this newly added layer is removed and the algorithm stops growing the topology.

## 6. The Common Frameworks

In addition to the proposed taxonomy, most methods use some specific frameworks to improve their performance.

### 6.1. Attention Mechanism

The attention mechanism helps emphasize the inference related information. Based on the dimension where the attention mechanism is used, approaches are divided as self-attention methods and other attention methods.

**Figure 10:**
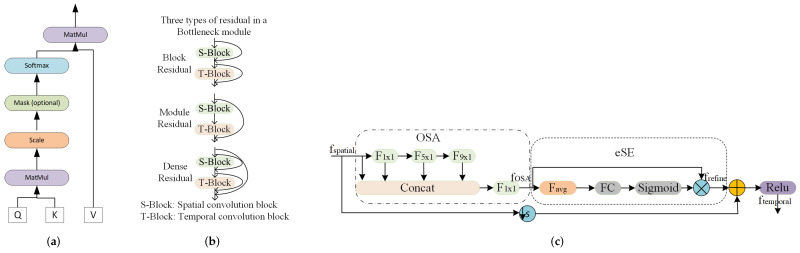
Examples of (**a**) self-attention [118], (**b**) the skip connections [119] and (**c**) the effective squeeze-excitation (eSE) block [120], where (**b**,**c**) are dense blocks.

In a general attention mechanism, there are three components, namely the *query* Q, the *key* K and the *value* V. After comparing the similarity between the *key* and the given *query*, one attention map is obtained and then employed on V so as to select discriminative values. The more similar are K and Q, the higher attention score that the corresponding *value* will have. Usually, the sigmoid functions or softmax functions are good candidates of similarity functions. One example of an attention mechanism is shown in Figure 10a.

#### 6.1.1. Self-Attention

Self-attention is the basic component in Transformers, commonly used in natural language processing (NLP). It is well-known for its capability of reweighting features. However, self-attention in NLP specifically includes an attention mechanism for the source domain or the target domain. Here, the definition of self-attention is extended. Since the model for HAR may not always have the target domain and source domain that occurs in NLP, as long as the features used for generating attention maps have the same meaning—in other words, are homogeneous—they are all regarded as self-attention. One common example is the attention maps between joints in skeleton graphs.

The first milestone of this idea is AGCN, which integrates attention with GNN by taking the attention map of the input joints as an added weight matrix. The attention map is then summed to the mask matrix, which is also used in ST-GCN. The self-attention matrix is estimated between input joints. All of the weight matrices are summed together to re-balance the relation of joints. Methods, such as [66,73,93,114,121] also used the same idea.

W. Peng et al. [122] adopted self-attention on joints and measure the similarity with a Gaussian similarity function. J. Shi et al. [123] proposed using the self-attention mechanism to calculate the weighted sum of the values from all nodes so as to aggregate features from the entire graph and also use the gating mechanism to adjust the weight of self-attention.

Y.B. Fan et al. [113] used a cross-attention module that consists of a self-attention branch and a cross-attention branch. Apart from paying more attention to informative joints in the self-attention branch, the combination of these two branches also suppress the influence of joints that are less relevant to the context information. T. Ahmad et al. [124] proposed a self-attention graph pooling to retain local properties and graph structures while pooling.

C. Plizzari et al. [125] highlighted a spatiotemporal Transformer network (ST-TR). In this model, the Spatial Self-Attention module (SSA) was used to understand intra-frame interactions between different body parts. They also adopted a Temporal Self-Attention module (TSA) to model inter-frame correlations.

W. Li et al. [126] proposed using self attention in spatial, temporal and channel dimension, which takes the features after global average pooling and max pooling as the original features, after one-dimensional convolution operation, they are regarded as the *query* and the *key* and then used softmax to calculate the attention map.

Instead of simple self-attention, such as what AGCN used, J. Xie et al. [127] integrated channel attention module (CAM) into their Vertex Attention Mechanism (VAM) to extract the global co-occurrence features of actions. The CAM generates channel weights by performing a fast 1DCNN in adaptive kernel size. This aggregated feature is used to generate attention map and later is added to sub-adjacency matrices. Finally, the complete summation of adjacency matrices is applied on the input node features.

#### 6.1.2. Other Attention Mechanism

Instead of calculating the relation between homogeneous features in self-attention, other attentions work on multi-domains, including spatial attention, temporal attention, channel attention and auxiliary data attention.

##### Spatial Attention

- Joint-level
  The joint-level spatial attention is the most used attention module, since it helps to re-weight joints and emphasize those task-informative joints. This is especially helpful in discovering the long-distance dependency.
  For each channel, Y.X. Chen et al. [85] converted the the relationship between local motion pattern and global motion pattern to an attention map, where the local features come from the rescaled graph and the global features come from the original skeleton graph. This can be regarded as a channel-separated joint-wise attention.
  To adaptively weight skeletal joints for different human actions, C. Li et al. [88] set a dynamic attention map that works on the features from spectral GCN. This attention map varies according to the spectral GCN features and different actions, and is used to weight nodes.
  S. Xu et al. [92] took the point-level hidden states captured by LSTM, as the *key* and the *query*. For different people, an attention map is captured to select informative joints.
  X.L. Ding et al. [96,128] used the attention graph interaction module, designed to pay different levels of attention to different joints and connections. The attention map is trained together with other parameters.
  The other one follows the popular recipe while using LSTM, which emphasizes the informative hidden state extracted by LSTM cell. For example, C. Si et al. [89] integrated the attention operation in LSTM cells. They take the weighted summation of all nodes’ hidden states as the *query*, and use sigmoid similarity function to select discriminative spatial information so that to enhance the information from key joints.
- Part-level
  Some researchers argue that the attention mechanism on joints is too localized and fails to detect the intra-part relation (global topologically relations).
  Since each action comprises of multiple interactions that happen in different parts, G. Zhang et al. [129] adopted multi-heads attention, which will generate multiple attention maps so as to focus on different parts. The attention model identifies key joints of every action by introducing two regularization terms, spatial diversity and local continuity. The spatial diversity is multi-head. It works by maximizing the distance between attention maps so as to focus on different parts. The local continuity is controlled by the attention map on graph’s Laplacian matrix.
  Y.F. Song et al. [119] concatenated the features of all parts and perform average pooling in temporal dimension, and then pass them through a fully connected layer with a BatchNorm layer and a ReLU function. Subsequently, five fully connected layers are adopted to calculate the attention matrices and a softmax function is utilized to determine the most essential body parts.
  Q.B. Zhong et al. [109] emphasized the joints with more motions and propose a novel local posture motion-based attention module (LPM-TAM) to filter out low motion information in temporal domain. This operation helps improve the ability of motion-related feature extraction. The attention map of skeleton sequence in the spatiotemporal graph is represented by the attention of local limbs estimated in temporal dimension.

##### Temporal Attention

Some approaches argue to use the temporal attention to select the most informative frames or joints that are instructive in temporal dimension. N. Heidari et al. [130] proposed a temporal attention module (TAM) to increase the efficiency in skeleton-based action recognition. It selects the most informative skeletons of an action, in other words, skeletons corresponding to the top $T^{\prime }$ highest attention values at the shallow layers of the network.

Most papers [73,74,97,109,126] calculated the temporal attention map with the most popular recipe, which adopts a sequence consisted of a pooling layer, a fully connection (FC) layer or one 1DCNN layer, followed by Relu or softmax activation functions. For example, L. Shi et al. [73] performed attention by 1DCNN. They first conducted average pooling for features and then processed the result by 1DCNN, and the attention map was calculated by softmax.

H.Y. Yang et al. [74] proposed a temporal and channel-wise attention (TCA) module, among which, the *query* is the node features after temporal and joint-wise global pooling, while the *key* is the node features after channel and joint-wise global pooling. The later follows the common recipe of attention. Q.B. Zhong et al. [109] processed the motion feature map by local posture motion-based temporal attention module and further by local posture motion-based channel attention module, with an aim at selecting the strongest discriminative representations between different posture movements.

##### Channel Attention

Methods, such as [49,73,74,100,120,126,127,131], assumed that features in different channels have various importance, and thus they attempted to balance the importance of each channel while inferring, known as channel-wise attention.

In [49], channel-wise attention was adopted in both GCN and gated CNN for information filtering. Squeeze-and-excitation (SE) block is the specific channel wise attention mechanism and was used after their action-specific graph convolutional module and gated convolution.

##### Auxiliary Data Attention

In addition to the attention on skeleton graphs, some also include attention on auxiliary data with the hope of completing the information that graph skeletons may be lack of. T. Ahmad et al. [132] used attention on RGB images to generate attention masks. These masks will be used on skeletons to pick attention joints.

### 6.2. Dense Block

The dense block attempts to compress features and also preserve gradients. There are mainly two types of dense block structures. One is the SE block [120], working on channel dimension, which performs a channel-wise attention for adaptive aggregation along the channel dimension. The other one is skip connection, also known as residual connection, which adds more connections between hidden states. One well-known example of skip connection is ResNet. It proposes to pass residual features by shortcut passing.

#### 6.2.1. Skip Connection

The skip connection is in fact a shortcut operation while passing information. By doing so, there are at least two advantages: one is that shortcuts help preserve gradients, preventing gradient vanishing after a long sequence and also ensure stable training. The other is that by carefully designing skip steps, one can control the dependencies along the temporal dimension both locally and adaptively. One example is demonstrated in Figure 10b.

The first benefit has been proven by numerous papers using CNN or RNN. Methods, such as [51,61,67,91,107,110,113,120,121,122,123,126,128,132,133,134,135], all follow the basic architecture of skip connection. The second benefit was also discovered by multiple papers.

C.Y. Si et al. [136] designed a skip-clip connection by adding shortcuts between the final hidden states from each clip and also between the adjacent skip-clip LSTM layers. In this way, gradients along adjacent clips and gradients among each frame in one clip are all taken care of. Paper [62] used the same idea. Some designed a more complex connection strategy to pass dependencies in multi-paths.

Y.F. Song et al. [119] (Figure 10b) proposed three types of skip connections, namely block residual, module residual and dense residual. The block residual, which adds connections between spatial-block and temporal-block, are regarded as the basic component of their modules. The module residual adds connections between modules, and the dense residual combines the connections in block residual and module residual.

Some researchers exploited the benefits of skip connections in different aspects. Xia. H et al. [137] used skip connections to fuse the information of the previous hop and the information of the next hop to collect information in different spatial scales. K. Papadopoulos et al. [112] preserved the information of short-term dependencies by skip connections.

#### 6.2.2. Squeeze-and-Excitation Block

Methods with SE block highlight the non-homogeneous features in each channel and attempt to squeeze features by pooling. One example of effective SE (eSE) is demonstrated in Figure 10c. F. Li et al. [120] concatenate consecutive layers by a one-shot aggregation (OSA) and the effective squeeze-excitation (eSE) block, an improved version of SE, which well balances the performance and efficiency. The eSE explores the interdependency between the output channels and squeezes the temporal and spatial dependencies.

Z.T. Zhang et al. [99] compress and extract multi-channel data through SE block to obtain the structure and feature weight. The weight is fused with the extracted features to obtain the spatial structure features.

### 6.3. Multi-Modalities

**Figure 11:**
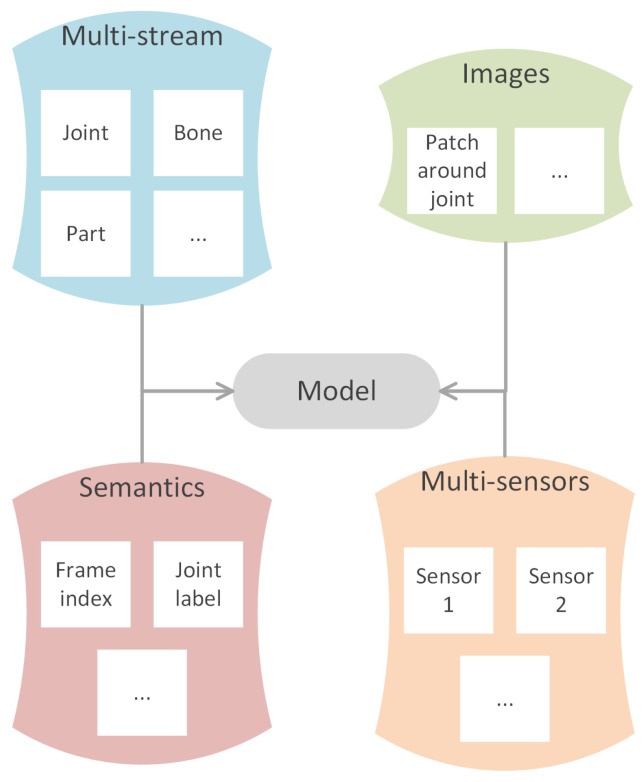
The candidates for a multi-modalities framework.

Multi-modalities is a very common framework using data in various domains. It can greatly help to improve the efficiency of a method since the weaknesses of one modality can be compensated by the strengths of another. The summary of possible candidates for modalities is shown in Figure 11.

#### 6.3.1. Multi-Stream

Multi-stream is the most commonly used structure in skeleton-GNN-HAR, methods, such as [14,49,52,54,68,69,71,73,87,89,90,93,99,104,114,119,120,123,125,126,134,137,138,139] all use this framework. This framework utilizes different types of data, such as joint stream, bone stream, part stream, relative coordinates of the joints, temporal displacements. One example is shown in Figure 2. Different types of data make up the information that one single stream is lacking of; however, the more streams that one approach adopts, the higher the computation price is.

#### 6.3.2. Multi-Sensors

Usually the sensors are the same but are fixed at different positions to obtain a multi-perspective dataset. For example, Y. Jiang et al. [102] used two Kinect sensors, which were orthogonal to each other, to help extract skeleton graphs from different perspectives.

#### 6.3.3. Semantics

Semantics information is also helpful in enhancing the feature representation capability. For example, P. Zhang et al. [135] used frame index and joint index in cooperation with their model. The joint index is important since two joints of the same coordinates but different semantics would deliver very different information. The temporal information (frame index) is also important to distinguish actions, such as sitting down and standing up from a chair, which are different only in posture occurrence order along the temporal dimension.

#### 6.3.4. Images

J.M. Cai et al. [54] used Joint-aligned optical Flow Patches (JFP) to capture the local subtle motion around each joint. The extracted features are taken as pivotal joint-centered visual information. T. Ahmad et al. [132] used attention on RGB images to generate attention masks, which helps to pick attention joints.

### 6.4. Change Feature Space

**Figure 12:**
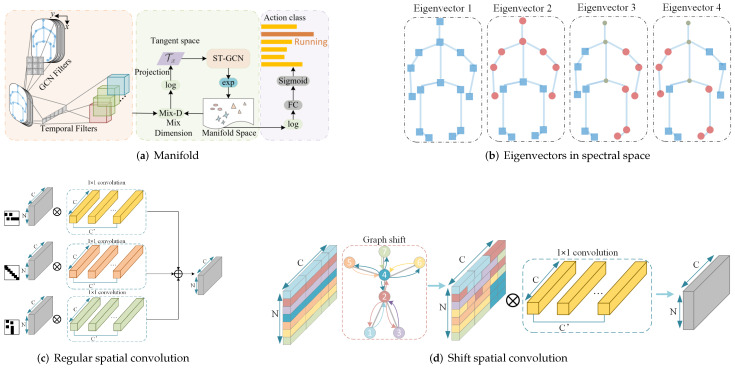
Examples of frameworks, where (**a**) [106], (**b**) [140] are examples, which change space, and (**c**,**d**) [141] are examples of neighbor convolution.

Rather than working in the original features space, some approaches attempted to explore a better modeling space for skeleton graphs, such as manifold and spectral space. Examples are shown in Figure 12a,b.

#### 6.4.1. Manifold

The idea of manifold is inspired by the non-Euclidean characteristics of skeleton graphs. W. Peng et al. [106] define their model on a Riemann manifold, which they argue is more suitable to model the graph data. Their model is built via the Poincaré geometry to better model the latent anatomy of the data structure.

#### 6.4.2. Spectral Space

One common idea of spectral space approaches is spectral GCN. Some implementations of spectral GCN are based on Chebyshev expansion. For example, methods [88,116] all adopt *k*-order Chebyshev expansion. J. kao et al. [140] designed graph representations for motion data, which is implemented by performing Graph Fourier transformation (GFT) on Laplacian matrix.

T. Ahmad et al. [124] performed spectral sparsification by exploiting similarity of the original graph, which is in Laplacian quadratic form, and that of the sparsed graph. It aims at discarding some redundant information by eliminating noisy nodes and edges.

### 6.5. Neighbors Convolution

The ways of convolution decide the characteristics of the aggregated features. The traditional way is *k*-neighbors convolution, while the one-hop convolution used in ST-GCN is the specific case of *k*-neighbors convolution when k=1. Others consider to modify the basic weighted summation in traditional convolutions, targeting at containing more structural information. Examples are shown in Figure 12c,d.

#### 6.5.1. The *k*-Neighbors Convolution

This kind of convolution aggregates the features of the node itself and its neighbors. According to the order of neighbors, the module are classified as one-hop and multi-hop. The method ST-GCN [13] and related methods [29,96,110,111,112,113] all adopt one-hop convolution. Methods [55,107,128] change one-hop to multi-hop, since the *k*-order of neighbors will capture more local information around the center node and also preserve the structural information around it.

#### 6.5.2. Other Convolution

One example is shift-convolution [141]. During shift convolution, the input features are first shifted along one predefined direction along body parts and then are weightly aggregated by 1DCNN. The paper proposes two kinds of spatial shift graph operations for modeling spatial skeleton graph, namely local shift graph convolution and non-local shift graph convolution. The first one’s receptive field is specified with the physical structure of the human body, while the later one makes the receptive field of each node cover the full skeleton graph.

## 7. Datasets

### 7.1. Means for Collecting Datasets

At the beginning, the 3D skeletons were directly captured by retro-reflective markers (RRMs), which were adhered to a suit and taken as joints. For example, with the VICON (Oxford, UK), a tracker system comprising RGB cameras, infrared-sensitive cameras and markers, movements and the 3D coordinates of every marker can be captured. This offers exact joint coordinates but no depth maps. Also, the requirement of markers and tracker system makes capturing intrusive and only possible under laboratory context.

Afterwards, as the emerging of DL-based skeleton extractors, e.g., Staked Hourglass [142], Google PoseNet [143], and especially the popularity of ToF cameras (Kinect [144]), binocular disparity-based cameras (Intel Realsense [145]), the marker-suit and complex tracking system are no longer or less necessary. Though the extracted joint position might still be influenced by the environment, for example when the subject’s clothes look similar to the background, this makes non-intrusive extraction in real-life possible. In addition to the simulation dataset captured under laboratory conditions, video platforms, such as YouTube are excellent candidates for action datasets, since their users have shared lots of videos under various contexts. Videos collected in this way are closer to real cases where HAR methods will be applied and therefore will help to fill the incompleteness shown in simulation datasets. Apart from collecting data from scratch, existing datasets are free to be used to recreate a new dataset, such as the UOW LSC [146] dataset, which combines various existing datasets.

### 7.2. Dataset Taxonomy

**Figure A1:**
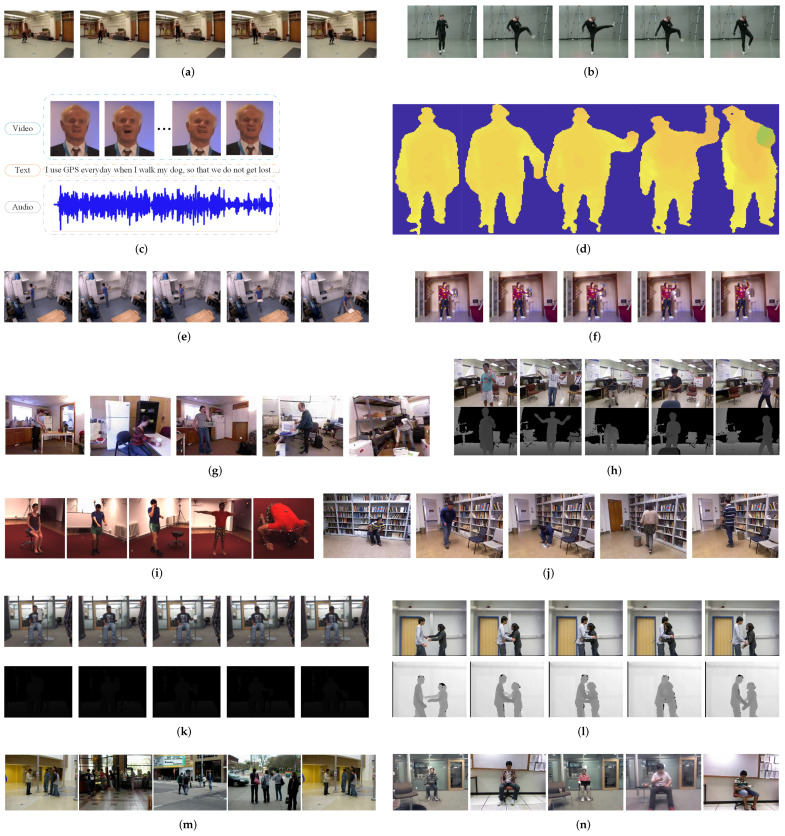
Examples of datasets. (**a**) CMU Mocap [167]; (**b**) HDM05 [155]; (**c**) IEMOCAP [156,197]; (**d**) MSR Action3D [147]; (**e**) TUM [157]; (**f**) Florence3D [159]; (**g**) CAD 60 and 120 [148,151]; (**h**) UT-Kinect [158]; (**i**) Human3.6M [168]; (**j**) N-UCLA [160]; (**k**) 3D Action Pairs [149]; (**l**) SBU Kinect Interaction [166]; (**m**) CA [171] and (**n**) ORGBD [152].

**Figure A2:**
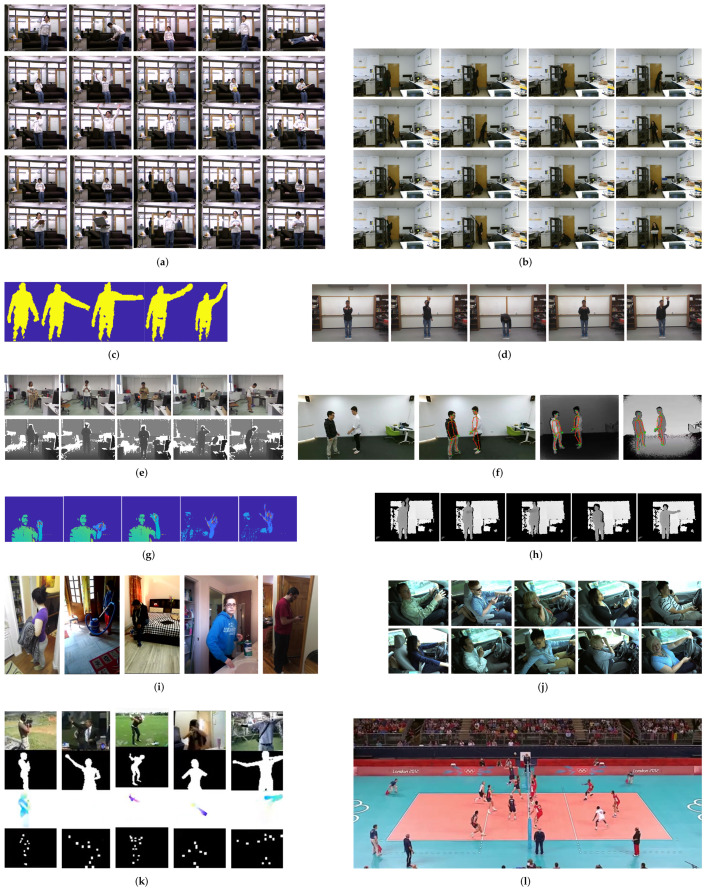
Examples of datasets. (**a**) MSR DailyActivity3D [150]; (**b**) UW-IOM [154]; (**c**) UWA3D Multiview [161,162]; (**d**) UTD MHAD [163]; (**e**) SYSU [153]; (**f**) NTU RGB+D [47]; (**g**) SHREC’17 [165]; (**h**) UOW LSC [146]; (**i**) Charades [173]; (**j**) StateFarm [175]; (**k**) J-HMDB [172] and (**l**) Volleyball [174].

**Figure A3:**
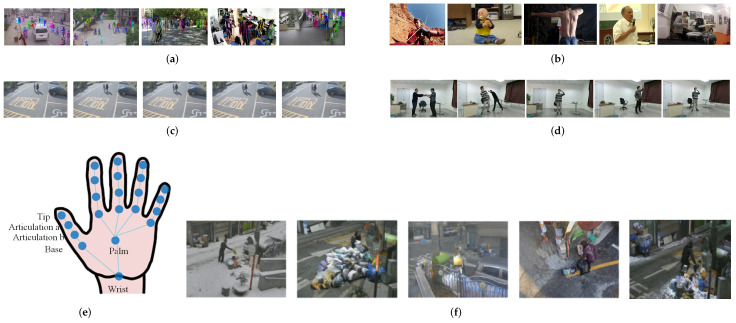
Examples of datasets. (**a**) HiEve [179]; (**b**) Kinetics [176]; (**c**) ICVL-4 [177]; (**d**) PUK-MDD [169]; (**e**) DHG-14/28 [164] and (**f**) IRD [177].

The datasets are grouped as simulation datasets and real-life datasets, each group is divided into single-subject actions, interacted actions and hybrid actions. The details of all mentioned datasets are summarized in Table A1 and Table A2 of the Appendix A, where Table A1 collects the basic information of the datasets, and Table A2 collects the activity list and related methods. The example of each dataset is illustrated in Figure A1, Figure A2 and Figure A3 of Appendix A.

#### 7.2.1. Simulation Datasets

##### Single-Subject Actions

MSR Action3D [147]: This dataset consists of 20 actions that interacting with game-consoles and each is performed three times by seven subjects. The depth maps are captured at about 15 frames per second (fps). The actions are classified into AS1, AS2 and AS3, where AS1 and AS2 intend to group actions with similar movements, and AS3 groups complex actions together. Depth maps and skeletons are offered without RGB videos.

CAD 60 [148]: RGB videos and aligned depth maps are captured by Kinect. Videos are taken in five different environments and the 12 unique activities (composed of several sub-activities) are performed by four actors. These actions cover the daily activities in office, kitchen, bedroom, bathroom and living room.

3D Action Pairs [149]: Six pairs of actions are carefully collected such that the two actions in each pair are similar in motions and shapes, with an aim to emphasize the importance of analyzing the shape and motion cues jointly during action recognition. Actions in every pair have similar trajectories and similar objects. Each action is performed three times by 10 actors. Depth sequences are offered but without skeletons.

MSR DailyActivity3D [150]: The dataset was captured by a Kinect. Sixteen activities in living room were recorded. Each of the ten subjects performs every activity twice: once in standing position, and once in sitting position. The depth maps, skeletons, and RGB videos are recorded; however, RGB videos and depth maps are not strictly synchronized.

CAD 120 [151]: After collecting 120 human–object interaction videos, this dataset annotates each video with the human skeleton tracks, object tracks, object affordance labels, sub-activity labels, and high-level activities. In total, four actors help to perform 10 sub-activities in 10 different contexts, e.g., making cereal, taking medicine, arranging objects.

ORGBD [152]: This dataset targets human–object interaction recognition based on RGBD videos. Seven actions were collected and performed by 36 subjects. The depth map, skeletons and RGB videos were offered.

SYSU [153]: It is a human–object interaction dataset performed by 40 subjects. There are 12 different activities, among which, each participant manipulated one of six different objects. The RGB videos, depth sequence and skeleton data were captured by Kinect in one view.

UW-IOM [154]: The University of Washington Indoor Object Manipulation dataset, designed for ergocentric risk detection, comprises videos from twenty subjects. They are classified into 17 action classes. Videos were recorded by Kinect at an average rate of 12 fps. Each participant performed to pick up six objects.

In addition to, the pure human–object datasets summarized above, others below in this section cover other single-subject action dataset (with or without objects).

HDM05 [155]: This dataset was introduced in 2005 and contains more than 70 motion classes performed by five actors. The resulting 3D trajectory data are stored in the C3D mocap file format. To capture videos, six RGB cameras and six infrared cameras were used within a VICON MX system.

IEMOCAP [156]: The Interactive Emotional Dyadic Motion Capture (IEMOCAP) database is an acted, multimodal and multispeaker dataset, collected in the SAIL lab at USC. Video, speech, motion capture of face and text transcription are all provided. During recording, 53 markers were attached to the face of the subjects, and they also wore wristbands, an extra marker on hand and headband. Finally, 9 emotions are labeled. Markers are captured by VICON optical tracker.

TUM [157]: The dataset is recorded in kitchen, tracked by four overhead cameras. The 28-joint skeletons were extracted and formated as BVH files. Since actions happening in a kitchen do not always involve every joint of the entire skeleton, the dataset is labeled separately. In nine actions, only the left hand is labeled. Two actions concentrate on the right hand, and two actions focus on the trunk of the skeleton.

UT-Kinect [158]: The videos were captured by a single stationary Kinect. There are 10 indoor actions, performed by 10 subjects. Each subject performs each action twice. The RGB videos, depth maps and skeleton are recorded.

Florence3D [159]: Captured by a Kinect camera, this dataset includes 9 daily activities. During capturing, 10 subjects were asked to perform the given actions.

N-UCLA [160]: It contains RGB, depth and human skeleton data, captured simultaneously by three Kinect cameras. Ten indoor daily actions are undertaken, each action is performed by 10 subjects.

UWA3D Multiview [161]: This dataset consists of 30 indoor daily activities from ten subjects’ performances at different scales, but all are taken in front view. This was captured by Kinect. The self-occlusions and high similarities in this dataset contribute to more challenges.

UWA3D Multiview Activity II [162]: In this dataset, 30 indoor actions are performed by 10 subjects from four different views at different scales. RGB videos, depth videos and skeletons are all provided.

UTD MHAD [163]: One Kinect camera and one wearable inertial sensor (WIS) are used to capture the RGB videos, depth maps and 20-joints skeletons. The dataset contains 27 indoor actions performed by eight subjects and repeated four times.

Additionally, there are some datasets created from other existing datasets.

UOW LSC [146]: By combing nine publicly available single-view RGB-D action datasets, this dataset with 94 actions was constructed. Data are all captured by Kinect, including datasets MSRAction3DExt, UTKinect, DailyActivity, ActionPair, CAD60, CAD120, G3D, RGBD-HuDa and UTD-MHAD. There are more viewpoints and subjects in this dataset. Actions include large motions of all body parts, e.g., spinal stretch, raising hands and jumping, and small movements of one part, e.g., head anticlockwise circle.

The following hand gesture datasets are also included into HAR action dataset, actions in these datasets are more similar and thus more challengeable since only the hand joints are recorded.

DHG-14/28 [164]: The DHG-14/28 dataset contains 14 gestures performed by 20 subjects in two ways: using one finger and using the entire hand. The captured sequences are labeled following their gestures, the number of fingers used, the performer and the trial. Each frame contains a depth image, the coordinates of 22 joints both in the 2D depth image space and in the 3D world space. The Intel RealSense short range depth camera was adopted as the capture device.

SHREC’17 [165]: The dataset contains 14 hand gestures sequences performed in two ways: using one finger and the entire hand. In these ways, each gesture is performed between 1 and 10 times by 28 participants. Each frame contains a depth image, the coordinates of 22 joints both in the 2D depth image space and in the 3D world space. The capturing device is Intel RealSense short range depth camera.

##### Interacted Actions

This part only discusses human–human interaction datasets.

SBU Kinect Interaction [166]: Using Kinect, this two-person interaction dataset was collected. Eight interaction actions are covered. Seven subjects perform activities in a laboratory and repeat. Apart from RGB videos and depth maps, the 15 joint skeletons were extracted by OpenNI with NITE middleware provided by PrimeSense.

##### Hybrid Actions

The dataset comprising hybrid actions include single-subject actions and interacted actions.

CMU Mocap [167]: The Carnegie Mellon University Motion Capture Database used 12 VICON infrared MX-40 cameras and 41 markers to build a 3D skeleton with 6 DOF at each joint. There are 144 participants who act both interaction motions and single subject motions. The actions are classified into 23 subcategories, covering human interaction, interaction with environment, locomotion, physical activities and sports and situations and scenarios.

Human3.6M [168]: The 3.6 Million accurate 3D Human poses are captured under four viewpoints and performed by 11 subjects. To capture 3D motions, four digital video cameras, one time-of-flight sensor and ten motion cameras were used. This dataset comprises 17 daily actions.

NTU RGB+D [47]: Built in 2016 and captured by three Kinect V2 cameras, NTU RGB+D contains 60 action classes and 56,880 video samples. RGB videos, depth map sequences, 3D skeletons, and infrared (IR) videos for each sample are provided. Each skeleton has 25 body joints. The 49 actions are preformed by a single subject, while 11 actions interacted.

PUK-MDD [169]: It contains 1076 long video sequences in 51 action categories (41 daily actions and 10 interactions), performed by 66 subjects in three Kinect camera views. The data including RGB videos, depth maps, Infrared images and skeletons are recorded under daily-life indoor context.

NTU RGB+D 120 [170]: Improved in 2019, this dataset extends NTU RGB+D by adding another 60 classes and another 57,600 video samples. The data type and the cameras are the same as in NTU RGB+D. Actions include 82 daily actions, 12 health-related actions (blowing nose, vomiting etc.) and 26 interacted actions (handshaking, pushing etc.).

#### 7.2.2. Real-Life Datasets

Datasets captured in real-life context are reliable and helpful for the generalization of methods, and also contain more challenges, such as occlusions, various environments. Collective Activity (CA) [171]: CA was proposed for group activity recognition. It contains 44 video clips, which are labeled with six individual action classes (null, crossing, walking, waiting, talking and queueing) and five group activity labels (crossing, walking, waiting, talking and queueing). Each clip has 10 frames, which was recorded by consumer hand-held digital cameras with varying view points.

J-HMDB [172]: Joint-annotated HMDB, an annotated subset of HMDB51 database, contains 21 single-person actions. Actions were collected from movies or the Internet. The 13-joint skeletons were extracted by 2D puppet model, while the model was constructed in 16 viewpoints.

Charades [173]: By distributing and crowdsourcing the entire process of video creation from script writing to video recording and annotation, Charades was collected as an indoor daily activity dataset, with 267 subjects performing 157 actions. Over 15% of the videos have more than one person. It can be used for egocentric vision research.

Volleyball [174]: This dataset was collected from publicly available YouTube volleyball videos. The 4830 frames, handpicked from 55 videos, were annotated with nine player action labels and eight team activity labels. The eight activities are right set, right spike, right pass, right winpoint, left pass, left spike and left set, while the nine actions are waiting, setting, digging, falling, spiking, blocking, jumping, moving and standing. Both RGB videos and bouding boxes are provided.

StateFarm [175]: This dataset was provided for a Kaggle competition in 2017, with an aim of classifying the RGB videos of drivers into 10 categories in order to check whether the driver is driving safely.

Kinetics [176]: It was built in 2017 by DeepMind, based on YouTube videos. In total, it contains 400 human action classes, and laterly was expanded to Kinetics-600, Kinetics-700. The actions include human–object interactions and human–human interactions. Skeleton dataset is not included.

ICVL-4 [177]: This is a subset of ICVL [178] real-time video surveillance dataset, with only object-related subsets being picked. Actions are divided into 13 categories, and each is performed by a single subject. Only the RGB videos and action labels are offered.

IRD (Illegal Rubbish Dumping) [177]: This human–object dataset comes from the post-processed videos by CCTV cameras. Actions are classified as garbage dumping and normal actions. All data were captured in unconstrained environments rather than indoor.

HiEve [179]: This dataset focuses on human-centric analysis in various crowd and complex events. Videos are collected from nine different scenes and 32 real-world video sequences are captured. Each subject in a video is annotated with a bounding box, 14 joint skeletons, human identities and human actions. In total, there are 14 action categories.

### 7.3. Performance

This part discusses the properties of datasets and the performance of the methods tested on them quantitatively and qualitatively. Precisely, we divide this section as the statistics of datasets, model performance, and hard activity cases. The datasets cover those summarized in Table A1, and the methods are those in Table A3.

#### 7.3.1. The Statistics of Datasets

**Figure 13:**
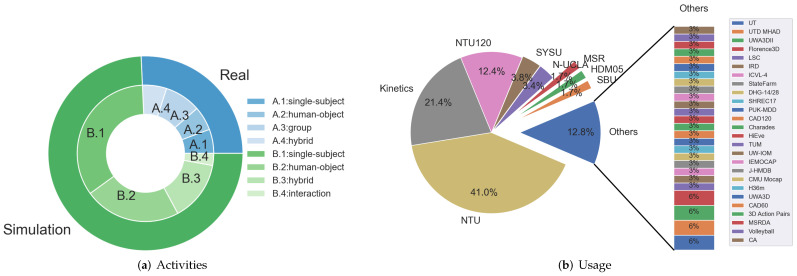
The properties of the cited datasets: (**a**) Action categories of every dataset, with single-subject actions (with or without objects), pure human–object actions, group activities, hybrid for single-subject and interaction actions, and interaction for pure human–human interactions. (**b**) How many methods are developed on each dataset. The methods are those listed in Table A3. The MSR stands for MSRAction3D, and MSRDA stands for MSR DailyActivity3D.

We classify the datasets according to action types, and count the methods evaluated on them. The results are shown in Figure 13a,b, respectively. From Figure 13a, most datasets are simulated rather than captured in real-life, and are performed by one subject. Only a few of the datasets focus on pure human–object or human–human interaction.

The top-eight most used datasets are NTU RGB+D, Kinetics, NTU RGB+D 120, SYSU, N-UCLA, MSRAction3D, HDM05 and SBU Kinect Iteraction (Figure 13b), among which, Kinetics is a real-life dataset, and others are simulated.

#### 7.3.2. Model Performance

The model performance can be estimated from the accuracy, model complexity and model size.

**Figure 14:**
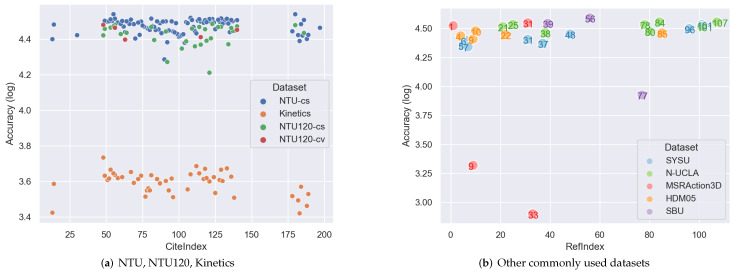
The performances of models (colored dots) on commonly used datasets: (**a**) The accuracy in logarithmic scale of cited methods (Table A3) on NTU RGB+D, NTU RGB+D 120 and Kinetics. (**b**) The performance (logarithmic) on SYSU, N-UCLA, MSRAction3D, HDM05 and SBU. Each method is denoted by its index Table A3, marked as RefIndex in the figure. The colors identity each dataset. In (**b**), the numbers around dots denote [12,13,29,56,59,61,69,72,74,75,83,85,87,88,89,90,92,97,110,115,135,136,138,141,182] respectively in ascending order.

- Accuracy
  The accuracy of methods is shown in Figure 14, with Figure 14a on NTU RGB+D, NTU RGB+D 120 and Kinetics, Figure 14b on SYSU, N-UCLA, MSRAction3D, HDM05 and SBU. Figure 14a demonstrates that the Kinetics dataset is more challengeable (scores are below 50% ) than simulated NTU, considering it is a real-life dataset and only provides RGB videos. Under real-life context, because of the occlusions, illuminations, complex environments etc., it is difficult to infer 3D skeleton graphs accurately. This huge challenge proves that the accurate 3D information is neccesary for skeleton-GNN-HAR.
  Moreover, it is clear that cross-subjects is more challengeable than cross-views, either on NTU or NTU120. This is because 3D skeletons are view-invariant, and under cross-view case, the 3D skeletons from different view points complement each other. When skeletons are from multiple subjects, the different sizes of subjects, separated clothes etc. all contribute to increase the recognition error.
  On other datasets, performance on MSRAction3D varies severely. MSRAction3D is challengeable because of the 3D information without RGB videos, and high interaction similarities. Specifically, ST-GCN [13] and ST-GCN-jpd [29] underperform others [12,69], where [12] used temporal pyramid, and [69] takes LSTM as the backbone. Methods that are good at temporal tracking outperform ST-GCN-based methods. This can be explained that the temporal evolution in ST-GCN is handled by CNN.
- Model Complexity and model size
  To show the complexity and model size of each approach, the floating point operations in Gigabytes FLOPs(G) for each action sample and the size of parameters in Megabytes are collected. Because none of the mentioned papers tested all methods under the same environment, for the same approach and same dataset, these statistics vary in different papers, due to the basic assumptions, devices, platforms, counting of multi-streams, resolutions etc. Therefore, for one single approach, if the statistics are different in multiple papers, we choose the maximum value. The model complexity FLOPs is measured on NTU RGB+D, collected from [54,57,80,117,121,141,180,181]. The model size is summarized from [54,55,57,82,85,117,121,135,180,181].

**Figure 15:**
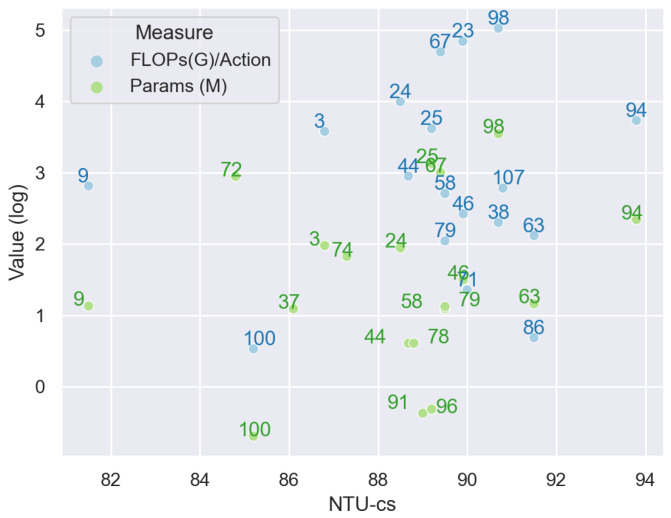
The logarithmic complexity and model size of the most popular methods (denoted as dots) performed on NTU cross-subjects. The text around each dot indicates the index of reference of the method. Green texts annotate the methods measured by ‘Params(M)’, and blue texts annotate ‘FLOPs (G)/Action’. The numbers around dots denote [13,14,50,54,55,57,59,62,73,77,80,82,85,86,89,110,116,117,121,127,130,135,141,183] respectively in ascending order. Number 94 [54] is a remarkable one, with both relatively low complexity and small model size.

According to [141], most GCN methods are over 15 FLOPs(G). Figure 15 illustrates the complexity and model size on NTU RGB+D in cross-subjects case. Dots that closed to the bottom right of the figure are the best models, with both lower complexity and lower model size but higher performance. JOLO-GCN [54] adopts multi-modalities– the Joint-aligned optical Flow Patches (JFP) to capture the local subtle motion around each joint, which proves the importance of local subtle motions.

#### 7.3.3. Hard Activity Cases

Despite many successes, there are hard activitiy cases that require further analysis. Unfortunately, only a few methods list the hard activities (less than 20% in our method set). We present them in this part.

- Similar single subject actions without objects
  For actions involving no objects, activities are mainly misclassified due to similar motion patterns with low pose resolution or inappropriate standardized axis coordinates.
  When actions only differ slightly around hand joints, the low hand pose resolution will increase the classification error. For instance, the NTU RBD+D dataset only records three joints for each hand, namely the wrist, the tip of the hand and the thumb. These joints are not enough to help distinguish actions with subtle movements around hands.
  Therefore, for actions with subtle hand movements, the NTU skeletons are less supportive in recognition. For example, Ref. [83] observed that actions, such as *rubbing two hands together and clapping*, are easily confused with each other on NTU, Ref. [71] misclassified *stand up* as *check time (from watch)* on NTU, Ref. [92] mistook *make victory sign* as *make ok sign*, *snapping fingers* as *make victory sign*. Ref. [64] misclassified *standing and walking* especially when the back of subjects faces the camera, which is due to the low pose resolution (missing of joints) caused by self-occlusions.
  Authors of [64] also discovered that actions like twisting are difficult because the standardized axis coordinates (Cartesian coordinates) erases the subtle rotation around the wrist.
- Similar single subject actions with objects
  Similar human–object interactions usually differ in the subtle movements of hands and have similar action trajectories.
  Generally, the errors are mainly caused by low pose resolution or lacking object information.
  Refs. [54,91] failed while classifying *reading, writing, playing with phone/tablet, and typing on a keyboard*. The authors argue these actions only differ for hand movements, while the skeletons provided by NTU RGB+D are less supportive for hand joints. Ref. [114] mentioned that when the body movements are not significant, and the sizes of the objects are relatively small, e.g., *counting money, and playing magic cube*, and the skeletons only provide three hand joints, the model can easily become confused. Ref. [89] also blamed the low NTU hands resolution, which leads to misclassify *reading* as *writing*, *writing* as *typing on keyboard*. As for distinguishing actions with subtle movements of two hands, such as *wearing a shoe, taking off a shoe*, Ref. [96] failed, and expects more precise hand joints to help. Similarly, Ref. [119] made mistakes on *reading and writing*, and holds the same opinion for fixing it.
  Ref. [71] misclassified *stapling book* into *cutting paper*, *counting money* into *playing magic cube*. The authors explained that this is because the information about objects is missing. This is supported by [83], where the authors observed that although actions, such as *drinking water and brushing teeth*, have similar motion patterns, the objects involved are different. Ref. [89] expects that their failure cases, such as *reading and writing*, can be erased by combining object appearance information.
- Human–human interaction
  In human–human interactions, one important reason of recognition errors given by the method set is occlusions.
  For instance, Refs. [82,111] observed that if important joints, such as right arms, are occluded, actions, such as handshaking cannot be inferred with the rest of the joints.

## 8. Challenges

Though the proposed approaches have shown their efficiency, there are still multiple challenges that call for further investigation.

### 8.1. Pose Preparation

#### 8.1.1. Real-Life Context

If datasets are collected under real-life context, the colorization, illumination, backgrounds etc. vary, caused by the complex and various environments, the capturing time, the exposure condition of capturing devices etc. These all contribute to increase the error of detected skeletons.

Occlusions is another natural problem in the real-life context, and is caused by surrounded objects, or by the subject himself (self-occlusions). Self-occlusions are common while doing actions, due to the interactions between body parts, such as playing basketball. The most rude way to deal with this situation is simply discarding those skeletons who have occlusions, which will lead to a huge loss. It is not only because of the cost of data acquisition but also the plenty of implicit information among occluded skeletons. One possible suggestion from [183] is RL-based pose refinement.

#### 8.1.2. Pose Resolution

The number of joints are different in different datasets. Normally, skeletons detected by DL methods, such as Openpose, PoseNet, have around 18 or 25 joints. Skeletons captured under simulation context, with RRM markers are more precise because of more joints. While distinguishing subtle movements of two hands, such as *clapping and rubbing two hands together*, more subtle hand joints are expected [71,96]. One possible solution is to mimic the hand gesture datasets during data capturing so as to provide more subtle hand joints.

#### 8.1.3. Pose Topology

Usually the pose topology is directly extracted from frames, following the physical human skeleton, and is fixed while inputing. Each joint has the similar weights during meassage passing, and follow the prefixed edges. However, Ref. [135] observed that different actions correspond to different informative joints. This indicates that there exist potential connections that are informative but missed by physical connections.

Ref. [137] discovered that the correlations of joints in learned spatial-temporal graph differ for different actions and different frames. Therefore, combining action information, spatial and temporal information with pose topology is one possible way to provide complementary information for recognition. The most common way is using self-attention to reweight joints.

### 8.2. View-Invariant

Human can easily recognize the same action, even when looking from different angles. However, combining the captured skeletons of one action observed from different views and recognizing them as the same action is more challengeable for machines, since one action appears quite differently if observed from another view. Features extracted from one viewpoint cannot be identified from another viewpoint accurately.

Currently, armed with RGBD cameras, reconstructing 3D human skeletons with captured depth videos is quite easy. Once the exact locations of skeleton joints are known, one can directly use estimated transformation matrix to make skeletons strictly view-invariant as standardized poses. Figure 14a demonstrates that 3D skeletons are helpful for HAR. However, when skeletons in different views are standardized to one pose, some partial relative motions among the original skeletons could be erased. For example, the action rotating wrists might be lost if all skeletons are standardized to one specific direction, such as facing the front.

Moreover, depth information is not always available. When only 2D skeletons and 2D videos in different views are provided, it is even more difficult to preserve view invariance due to the lack of 3D information.

### 8.3. Multiscale

#### 8.3.1. Multi Spatial Scale

Most approaches only extract spatial information at the joint scale, without considering feature extraction at multiple scales. However, for different actions, features just from joint scale are not subtle enough for accurate recognition. For example, actions cutting and writing all move hands and arms. If all joints are taken into consideration equally, rather than paying more attention to arms and hands, the result will under-perform since many local information will be ignored. Therefore, capturing local features without losing global features is critical. One hint to solve this is based on skeleton partition, such as part-based partition [57,64,86,95].

#### 8.3.2. Multi Temporal Scale

Temporal scale also contains implicit features, e.g., the actions race walking and walking are visually similar; however, if one compares the frames at different time scales, they are different. Moreover, the order of frames (timesteps) is also important when taking sitting down and standing up into consideration. To deal with this, some previous works performed dilated temporal convolution [55,84], while others simply sampled specific frames [104,137].

#### 8.3.3. Multi Subject Scale

There are two major problems caused by this situation, one is for interacted actions, how to balance features from inter subjects and features from intra subjects. Paper [92] offers a hint to perform attentioned LSTM in joint-level, person-level and scene-level so as to trade-off both the intra-subject and inter-subject information. The other is when there are numerous subjects, such as in the Kinetics dataset. From the performance of methods on NTU RGB+D (Figure 14a), the cross-subjects case is more challengeable compared with the cross-views case.

### 8.4. Multi-Modalities

#### 8.4.1. Multi-Modalities Fusion

Understanding video content precisely benefit from multimodalities. Ref. [123] proves that each modality (skeleton, audio, text) had its unique strengths, and believes that a better fusion strategy may improve performance. Ref. [73] clarifies the complementarity between the skeleton modality and the RGB modality. Ref. [113] argues that, except for RGB video, video captions can also provide context information.

However, the challenges in modalities fusion come from resolution, fusion strategy, alignment etc. Inspired approaches for solving these problems include [54], which proposes JFP (optical flow patches) sequences to capture the local subtle motion information, Ref. [184] that used speech texts, Ref. [185], which used context information, and AdaMML [186] adaptively selected which modality to use for each video segment.

#### 8.4.2. Inner Heterogeneous

From Section 3, we know that graph edges are heterogeneous if considering temporal edges, spatial edges etc. Normally, the GNN layer is used on the entire graph rather than treating these edges heterogeneously. Traditional methods, such as the recipe combining of GNN and LSTM, treat spatial edges and temporal edges separately; however, they ignore the inter relations of spatiotemporal data. Tackling the heterogeneous nodes or edges in graphs but without ignoring the original relations among them has created a large question for the scientific community.

For that case, X. Gao et al. [75] suggested distinguishing intra-frame and inter-frame. Precisely, joints in intra-frame are grouped as weakly connected and strongly connected joints, and their corresponding edges are further emphasized by two constant weights. Temporal edges in inter-frames are also assigned with two constant weights to distinguish edges between corresponding joints and edges between each point and the neighborhood of its correspondence in the adjacent frames.

### 8.5. Interactions

Most methods focus on single skeleton graph-based HAR. However, in addition to this simple case, there are two more complex cases: the human–human interaction and human–object interaction, errors among them are not the same [187].

#### 8.5.1. Human-Human Interaction

For human–human interactions, such as *hugging, kicking*, there are at least two skeletons in each frame. In some complex cases, such as *walking through zebra crossing*, more skeletons are obtained. Either grouping multiple skeletons to detect sub-activities or handling local and global features simultaneously all can be a problem.

Coping with this, papers [48,187,188] concentrate on relative features, paper [101] splits the motion model into single person and double person motion model, paper [115] attempted to transfer the knowledge between the two interacted skeletons by the teacher–student mechanism. Ref. [125] discovered the long-range relations on two people actions benefit from self-attention, because self-attention detects the correlation along the entire action. Ref. [189] used distance, orientation and O-space features to describe the relations between subjects.

#### 8.5.2. Human-Object Interaction

There are two main tasks in human–object interactions. One is object detection, the other is HAR according to the semantics of object detection. Human–objects interactions that have subtle movements of hands can be easily misidentified [83,91,92]. However, Ref. [61] observed that almost no skeleton motion and the differences are illustrated as human–object interaction.

Some existing papers give inspiration. S. Kim et al. [177] split the problem into two sub-problems and build two streams upon it, which are the human pose stream and the object-related pose stream. X. Shi et al. [190] treated the related objects as joint points and linked them to hands. Refs. [61,89] recommend to combine object appearance for similar movements, Ref. [119] preferred adding more hand joints while collecting datasets.

## 9. Conclusions

In this paper, we thoroughly analyzed skeleton graph-based HAR. Concretely, the types of skeleton graphs and the means to build graph structures were discussed. We proposed a new taxonomy that classifies the skeleton-GNN-based HAR into spatial approaches, spatiotemporal approaches and generated approaches. The most common frameworks were also summarized, including the attention mechanism, dense blocks, multi-modalities, changing feature space and neighbor convolution. Finally, the most frequently used datasets were also collected and described.

**Figure 16:**
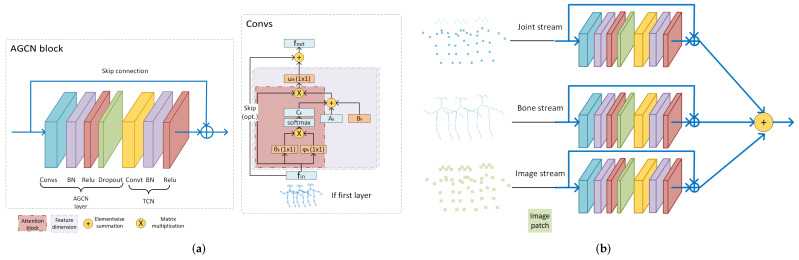
The common recipe for skeleton-GNN-based HAR: (**a**) [14] the attention plus skip connections, (**b**) a way to use multi-modalities with multi-stream.

Based on the previous discussion, the possible future directions are apparent. Usually, the built graphs are undirected graphs, and many researchers attempted to develop models based on ST-GCN or AGCN, the two milestones in this field. Additionally, the two frameworks (the attention mechanism plus skip connections and multi-stream with multi-modalities) were preferred. The details are shown in Figure 16.

Though numerous proposed methods have solved many problems, challenges from pose preparation, view-invariant, occlusion, multi-scale, multi-modalities and interactions remain to be solved. 

## Appendix A. Datasets

Table A1 presents a list of the relevant datasets for HAR with their main characteristics.

**Table A1:** The summary of datasets. ’RGB’ is RGB videos, ’IR’ is infrared sequences, ’D’ is depth maps, ’S’ is skeletons, ’PD’ is pose direction, ’OP’ is optical flow, ’WIS’ is wearable inertial sensor, ’TOF’ is time-of-flight sensor, ’RRM’ is retro-reflective markers, ’H’ is head movement, ’Script’ is dialog transcriptions, and ’M’ is mesh data.

Name	Sensors	Subjects	Views	Actions	Data	Year	Types
CMU Mocap, http://mocap.cs.cmu.edu/	Vicon	144	-	23	RGB+S	2003	Indoor simulation, including interaction.
HDM05, http://resources.mpi-inf.mpg.de/HDM05/	RRM	5	6	>70	RGB+S	2007	Indoor simulation
IEMOCAP, https://sail.usc.edu/iemocap/	Vicon	-	8	9	RGB+H + Script	2008	Emotion and speech dataset
CA, https://cvgl.stanford.edu/projects/collective/collectiveActivity.html	Hand held camera	-	-	5	RGB+PD	2009	Group activities
TUM, https://ias.in.tum.de/dokuwiki/software/kitchen-activity-data	-	-	4	9 (l),9 (r), 2 (t)	RGB+S	2009	Activities in kitchen
MSR Action3D, https://sites.google.com/view/wanqingli/data-sets/msr-action3d	-	10	1	20	D+S	2010	Interaction with game consoles
CAD 60, https://drive.google.com/drive/folders/1Z5ztMpeys5I0XfZn8J26-_6rqplweFGI	Kinect v1	4	-	12	RGB+D+S	2011	Human–object interaction
MSR DailyActivity3D, https://sites.google.com/view/wanqingli/data-sets/msr-dailyactivity3d	Kinect	10	1	16	RGB+D+S	2012	Daily activities in living room
UT-Kinect, http://cvrc.ece.utexas.edu/KinectDatasets/HOJ3D.html	Kinect v1	10	4	10	RGB+D+S	2012	Indoor simulation
Florence3D, https://www.micc.unifi.it/resources/datasets/florence-3d-actions-dataset/	Kinect v1	10	-	9	RGB+S	2012	Indoor simulation
SBU Kinect Interaction, https://www3.cs.stonybrook.edu/~kyun/research/kinect_interaction/index.html	Kinect	7	-	8	RBB+D+S	2012	Human–human interaction simulation
J-HMDB, http://jhmdb.is.tue.mpg.de/	HMDB51	-	16	21	RGB+S	2013	Annotated subset of HMDB51, 2D skeletons, real-life
3D Action Pairs, http://www.cs.ucf.edu/~oreifej/HON4D.html	Kinect v1	10	1	12	RGB+D+S	2013	Each pair has similarity in motion and shape
CAD 120, https://www.re3data.org/repository/r3d100012216	Kinect v1	4	-	10+10	RGB+D+S	2013	Human–object interaction
ORGBD, https://sites.google.com/site/skicyyu/orgbd	-	36	-	7	RGB+D+S	2014	Human–object interaction
Human3.6M, http://vision.imar.ro/human3.6m/description.php	Laser scanner, TOF	11	4	17	RGB+S+M	2014	Indoor simulation, meshes
N-UCLA, http://wangjiangb.github.io/my_data.html	Kinect v1	10	3	10	RGB+D+S	2014	Daily action simulation
UWA3D Multiview, https://github.com/LeiWangR/HDG	Kinect v1	10	1	30	RGB+D+S	2014	Different scales, including self-occlusions and human–object interaction.
UWA3D Multiview Activity II, https://github.com/LeiWangR/HDG	Kinect v1	10	4	30	RGB+D+S	2015	Different views and scales, including self-occlusions and human–object interaction.
UTD-MHAD, https://personal.utdallas.edu/~kehtar/UTD-MHAD.html	Kinect v1 + WIS	8	1	27	RGB+D+S	2015	Indoor single-subject simulation
NTU RGB+D, https://rose1.ntu.edu.sg/dataset/actionRecognition/	Kinect V2	40	80	50 + 10	RGB+IR +D+S	2016	Simulation, including human–human interaction
Charades, https://prior.allenai.org/projects/charades	-	267	-	157	RGB+OP	2016	Real-life daily indoor activities
UOW LSC, https://sites.google.com/view/wanqingli/data-sets/uow-largescale-combined-action3d	Kinect	-	-	94	RGB+D+S	2016	Combined dataset
StateFarm, https://www.kaggle.com/c/state-farm-distracted-driver-detection/data	Kaggle competition	-	-	10	RGB	2016	Real driving videos
DHG-14/28	Intel RealSense	20	-	14/28	RGB+D+S	2016	Hand gestures
Volleyball, https://github.com/mostafa-saad/deep-activity-rec	YouTube volleyball	-	-	9	RGB	2016	Group activities, volleyball
SYSU, https://www.isee-ai.cn/~hujianfang/ProjectJOULE.html	Kinect v1	40	1	12	RGB+D+S	2017	Human–object interaction
SHREC’17, http://www-rech.telecom-lille.fr/shrec2017-hand/	Intel RealSense	28	-	-	RGB+D+S	2017	Hand gestures
Kinetics, https://deepmind.com/research/open-source/kinetics	YouTube	-	-	400	RGB	2017	Real life, including human–object interaction and human–human interaction
PUK-MDD, https://www.icst.pku.edu.cn/struct/Projects/PKUMMD.html	Kinect	66	3	51	RGB+D+ IR+S	2017	Daily action simulation, including interactions.
ICVL-4, https://github.com/ChengBinJin/ActionViewer	-	-	-	13	RGB	2018	Human–object action in real-life, a subset of ICVL.
UW-IOM, https://data.mendeley.com/datasets/xwzzkxtf9s/1	Kinect	20	-	17	RGB+D+S	2019	Indoor object manipulation
NTU RGB+D 120, https://rose1.ntu.edu.sg/dataset/actionRecognition/	Kinect V2	106	155	94 + 26	RGB+IR + D+S	2019	Simulation, including human–human interaction
IRD	CCTV	-	-	2	RGB	2019	Illegal rubbish dumping in real life
HiEve, http://humaninevents.org/	-	-	-	14	RGB+S	2020	Multi-person events under complex scenes

Table A2 summarizes the action list and methods performed on each dataset. The most popular compared methods across datasets are [13,14,50,54,55,57,59,62,73,77,80,82,85,86,89,110,116,117,121,127,130,135,141,183], among which, Ref. [54] is a remarkable one on NTU RGB+D cross-subject, with both low complexity and small model size but good performance.

**Table A2:** The summary of datasets and methods.

Name	Papers	Action List
CMU Mocap	[95]	Human Interaction, Interaction With Environment, Locomotion, Physical Activities + Sports, Situations + Scenarios
HDM05	[56,61,74,88]	Walk, Run, Jump, Grab and Deposit, Sports, Sit and Lie Down, Miscellaneous Motions
IEMOCAP	[123]	Anger, Happiness, Excitement, Sadness, Frustration, Fear, Surprise, Other and Neutral State
CA	[115]	Null, Crossing, Wait, Queueing, Walk, Talk
TUM	[64]	Set the Table, Transport Each Object Separately as Done by an Inefficient Robot, Take Several Objects at Once as Humans Usually Do, Iteratively Pick Up and Put Down Objects From and to Different Places
MSR Action3D	[12,29,69,140]	High Arm Wave, Horizontal Arm Wave, Hammer, Hand Catch, Forward Punch, High Throw, Draw X, Draw Tick, Draw Circle, Hand Clap, Two Hand Wave, Side Boxing, Bend, Forward Kick, Side Kick, Jogging, Tennis Swing, Tennis Serve, Golf Swing, Pick Up + Throw
CAD 60	[29]	Still, Rinse Mouth, Brush Teeth, Wear Contact Lenses, Talk on Phone, Drink Water, Open Pill Container, Cook (Chop), Cook (Stir), Talk on Couch, Relax on Couch, Write on Whiteboard, Work on Computer
MSR DailyActivity3D	[115]	Drink, Eat, Read Book, Call Cellphone, Write on a Paper, Use Laptop, Use Vacuum Cleaner, Cheer Up, Sit Still, Toss Paper, Play Game, Lay Down on Sofa, Walk, Play Guitar, Stand Up, Sit Down
UT-Kinect	[12,140]	Walk, Sit Down, Stand Up, Pick Up, Carry, Throw, Push, Pull, Wave Hands, Clap Hands
Florence3D	[12,88]	Wave, Drink From a Bottle, Answer Phone, Clap, Tight Lace, Sit Down, Stand Up, Read Watch, Bow
SBU Kinect Interaction	[83,115,182,187]	Approach, Depart, Push, Kick, Punch, Exchange Objects, Hug, and Shake Hands
J-HMDB	[124]	Brush Hair, Catch, Clap, Climb Stairs, Golf, Jump, Kick Ball, Pick, Pour, Pull-Up, Push, Run, Shoot Ball, Shoot Bow, Shoot Gun, Sit, Stand, Swing Baseball, Throw, Walk, Wave
3D Action Pairs	[29]	Pick Up a box/Put Down a Box, Lift a box/Place a Box, Push a chair/Pull a Chair, Wear a hat/Take Off a Hat, Put on a backpack/Take Off a Backpack, Stick a poster/Remove a Poster.
CAD 120	[67]	Make Cereal, Take Medicine, Stack Objects, Unstack Objects, Microwave Food, Pick Objects, Clean Objects, Take Food, Arrange Objects, Have a Meal
ORGBD	[115]	Drink, Eat, Use Laptop, Read Cellphone, Make Phone Call, Read Book, Use Remote
Human3.6M	[95]	Conversations, Eat, Greet, Talk on the Phone, Pose, Sit, Smoke, Take Photos, Wait, Walk in Various Non-Typical Scenarios (With a Hand in the Pocket, Talk on the Phone, Walk a Dog, or Buy an Item)
N-UCLA	[59,87,89,90,92,94,138,141]	Pick Up With One Hand, Pick Up With Two Hands, Drop Trash, Walk Around, Sit Down, Stand Up, Donning, Doffing, Throw, Carry
UWA3D Multiview	[29]	One Hand Wave, One Hand Punch, Sit Down, Stand Up, Hold Chest, Hold Head, Hold Back, Walk, Turn Around, Drink, Bend, Run, Kick, Jump, Mope Floor, Sneeze, Sit Down (Chair), Squat, Two Hand Wave, Two Hand Punch, Vibrate, Fall Down, Irregular Walk, Lie Down, Phone Answer, Jump Jack, Pick Up, Put Down, Dance, Cough
UWA3D Multiview Activity II	[29,94]	One Hand Wave, One Hand Punch, Two Hand Wave, Two Hand Punch, Sit Down, Stand Up, Vibrate, Fall Down, Hold Chest, Hold Head, Hold Back, Walk, Irregular Walk, Lie Down, Turn Around, Drink, Phone Answer, Bend, Jump Jack, Run, Pick Up, Put Down, Kick, Jump, Dance, Mope Floor, Sneeze, Sit Down (Chair), Squat, Cough
UTD-MHAD	[69,105]	Indoor Daily Activities. Check https://personal.utdallas.edu/~kehtar/UTD-MHAD.html for details
NTU RGB+D	[13,14,48,49,50,51,52,53,54,55,56,57,59,60,61,62,63,65,66,68,69,71,72,73,74,75,76,77,78,79,80,81,82,83,84,85,86,87,88,89,90,91,92,93,94,95,96,97,99,100,101,102,103,104,105,106,107,109,110,111,112,113,114,115,116,117,119,120,121,122,124,125,126,127,128,129,130,131,132,133,134,135,136,137,138,139,141,182,183,187,191,192,193,194,195,196]	https://rose1.ntu.edu.sg/dataset/actionRecognition/
Charades	[67]	https://prior.allenai.org/projects/charades
UOW LSC	[88]	Large Motions of All Body Parts, E.g., Spinal Stretch, Raising Hands and Jumping, and Small Movements of One Part, E.g., Head Anticlockwise Circle. Check https://sites.google.com/view/wanqingli/data-Sets/uow-Largescale-Combined-Action3d for details.
StateFarm	[192]	Safe Drive, Text-Right, Talk on the Phone-Right, Text-Left, Talk on the Phone-Left, Operate the Radio, Drink, Reach Behind, Hair and Makeup, Talk to Passenger
DHG-14/28	[70]	Grab, Tap, Expand, Pinch, Rotate Clockwise, Rotatr Couter Clockwise, Swipe Right, Swipe Left, Swipe Up, Swipe Down, Swipe X, Swipe V, Swipe +, Shake
Volleyball	[115]	Wait, Set, Dig, Fall, Spike, Block, Jump, Move, Stand
SYSU	[62,69,72,75,85,90,97,135,136]	Drink, Pour, Call Phone, Play Phone, Wear Backpacks, Pack Backpacks, Sit Chair, Move Chair, Take Out Wallet, Take From Wallet, Mope, Sweep
SHREC’17	[70]	Grab, Tap, Expand, Pinch, Rotate Clockwise, Rotatr Couter Clockwise, Swipe Right, Swipe Left, Swipe Up, Swipe Down, Swipe X, Swipe V, Swipe + and Shake
Kinetics	[13,14,48,49,50,51,52,53,54,55,57,60,66,68,71,73,76,77,78,79,80,84,86,91,93,95,96,99,107,109,113,116,117,120,121,122,124,127,128,130,131,133,134,137,139,183,191,192,195,196]	https://deepmind.com/research/open-Source/kinetics
PUK-MDD	[132]	41 Daily + 10 Interactions. Details are shown in https://www.icst.pku.edu.cn/struct/Projects/PKUMMD.html
ICVL-4	[177]	Sit, Stand, Stationary, Walk, Run, Nothing, Text, and Smoke, Others
UW-IOM	[64]	17 Actions as a Hierarchy Combination of four Tiers: Whether the Box or the Rod Is Manipulated, Human Motion (Walk, Stand and Bend), Captures the Type of Object Manipulation if Applicable (Reach, Pick-Up, Place and Hold) and the Relative Height of the Surface Where Manipulation Is Taking Place (Low, Medium and High)
NTU RGB+D 120	[48,52,54,55,59,63,80,82,91,92,99,103,106,110,112,114,119,120,121,122,124,125,126,135,137,138,141,187,194]	82 Daily Actions (Eating, Writing, Sitting Down etc.), 12 Health-Related Actions (Blowing Nose, Vomiting etc.) and 26 Mutual Actions (Handshaking, Pushing etc.).
IRD	[177]	Garbage Dump, Normal
HiEve	[188]	Walk-Alone, Walk-Together, Run-Alone, Run-Together, Ride, Sit-Talk, Sit-Alone, Queuing, Stand-Alone, Gather, Fight, Fall-Over, Walk-Up-Down-Stairs and Crouch-Bow

Examples of each dataset are listed in Figure A1, Figure A2 and Figure A3.

## Appendix B. Methods

Table A3 shows a list of the most important methods for HAR with their main characteristics and accuracy.

**Table A3:** The summary of methods and their accuracy. The accuracy on each dataset is top-1. AE is Autoencoder, SVM is support vector machine, LSTM is long-short term memory network, TCN is temporal convolution network, RL is reinforcement learning, CAM is class activation maps, GFT is graph fourier transform, SE is squeeze-excitation block, TCN is temporal convolution, conv. is convolution, PGNs is Pyramidal GCNs, and FV is Fisher Vector.

				Datasets				
Name	Code	Year	Details	Kinetics		NTU RGB+D		NTU RGB+D 120
					CV	CS	CV	CS
GCN [12]	-	2017	GCN+SVM	-	-	-	-	-
STGR [76]	-	2018	Concatenate spatial router and temporal router	33.6	92.3	86.9	-	-
AS-GCN [77]	Github	2018	AE, learn edges	34.8	94.2	86.8	-	-
$A^{2}\mathrm{GNN}$ [88]	-	2018	GCN+LSTM, adaptively weighting skeletal joints	-	82.8	72.74	-	-
GR-GCN [75]	-	2018	LSTM	-	94.3	87.5	-	-
SR-TSL [136]	-	2018	GCN+clip LSTM	-	92.4	84.8	-	-
DPRL [72]	-	2018	RL	-	89.8	83.5	-	-
BPLHM [139]	-	2018	Edge aggregation	33.4	91.1	85.4	-	-
ST-GCN [13]	Github	2018	ST-GCN	30.7	88.3	81.5	-	-
PB-GCN [61]	Github	2018	Skip connection, subgraphs, graphs are overlapped	-	93.2	87.5	-	-
3s RA-GCN [111]	Github	2019	ST-GCN backbone, softmax for CAM	-	93.5	85.9	-	-
AR-GCN [96]	-	2019	Skip connection, BRNN + attentioned ST-GCN, spatial and temporal attention	33.5	93.2	85.1	-	-
GVFE+AS-GCN with DH-TCN [112]	-	2019	ST-GCN-based, dilated temporal CNN, skip connection	-	92.8	85.3	-	78.3
BAGCN [53]	-	2019	LSTM	-	96.3	90.3	-	-
[101]	-	2019	ST-GCN	-	89.6	82.6	-	-
[140]	-	2019	GFT	-	-	-	-	-
OHA-GCN [177]	-	2019	Human–object, frame selection + GCN	-	-	-	-	-
AM-STGCN [191]	-	2019	Attention	32.9	91.4	83.4	-	-
[192]	-	2019	GCN	30.59	88.87	80.66	-	-
[70]	-	2019	Add edges, hand gestures	-	-	-	-	-
GCN-HCRF [87]	-	2019	HCRF, directed message passing	-	91.7	84.3	-	-
Si-GCN [56]	-	2019	Structure induced part-graphs	-	89.05	84.15	-	-
4s DGNN [50]	Github	2019	Directed graph	36.9	96.1	89.9	-	-
2s-AGCN [14]	Github	2019	Two stream, attention	36.1	95.1	88.5	-	-
2s AGC-LSTM [89]	-	2019	Attention	-	95.0	89.2	-	-
SDGCN [133]	-	2019	Skip connection	-	95.74	89.58	-	-
JRIN-SGCN [78]	-	2019	Adjacent inference	35.2	91.9	86.2	-	-
JRR-GCN [79]	-	2019	RL for joint-relation-reasoning	34.8	91.2	85.89	-	-
3heads-MA-GCN [129]	-	2019	Multi-heads attention	-	91.5	86.9	-	-
GC-LSTM [98]	-	2019	LSTM	-	92.3	83.9	-	-
Bayesian GC-LSTM [69]	-	2019	Bayesian for the parameters of GC-LSTM	-	89	81.8	-	-
RGB + skeleton [113]	-	2020	Cross attention (joints + scenario context information), ST-GCN backbone	39.9	89.27	84.23	-	-
ST-GCN-jpd [29]	-	2020	ST-GCN backbone	-	88.84	83.36	-	-
[132]	-	2020	Skip connection, attention to select joints	-	-	90.7	-	-
2s-FGCN [68]	-	2020	Fully connected graph	36.3	95.6	88.7	-	-
2s-GAS-GCN [49]	-	2020	Gated CNN, channel attention	37.8	96.5	90.4	-	86.4
SGP-JCA-GCN [85]	-	2020	Structure-based graph pooling, learn edges between human parts	-	93.1	86.1	-	-
4s Shift-GCN [141]	Github	2020	Cheap computation, shift graph convolution	-	96.5	90.7	85.9	87.6
STG-INs [83]	-	2020	LSTM	-	88.7	85.8	-	-
MS-AGCN [114]	-	2020	Multistream, AGCN backbone	-	95.8	90.5	-	-
MSGCN [65]	-	2020	Attention+SE block, scaled by part division	-	95.7	88.8	-	-
Res-split GCN [51]	-	2020	Directed graph, skip connection	37.2	96.2	90.2	-	-
Stacked-STGCN [67]	-	2020	Hourglass, human–object-scene nodes	-	-	-	-	-
2s-PST-GCN [117]	-	2020	Find new topology	35.53	95.1	88.68	-	-
2s-ST-BLN [81]	-	2020	Symmetric spatial attention, symmetric of relative positions of joints	-	95.1	87.8	-	-
4s-TA-GCN [130]	-	2020	Skip connection, temporal attention	36.9	95.8	89.91	-	-
DAG-GCN [100]	-	2020	Joint and channel attention, build dependence relations for bone nodes	-	95.76	90.01	82.44	79.03
LSGM+GTSC [97]	-	2020	LSTM, feature calibration, temporal attention	-	91.74	84.71	-	-
VT+GARN (Joint&Part) [94]	-	2020	View-invariant, RNN	-	-	-	-	-
[102]	-	2020	Skeleton fusion, ST-GCN backbone	-	-	82.9	-	-
4s-EE-GCN [120]	-	2020	One-shot aggregation, CNN	39.1	96.8	91.6	-	87.4
MS-ESTGCN [134]	-	2020	Spatial conv. + temporal conv.	39.4	96.8	91.4	-	-
EN-GCN [193]	-	2020	Fuse edge and node	-	91.6	83.2	-	-
MS TE-GCN [194]	-	2020	GCN+1DCNN as TCN	-	96.2	90.8	-	84.4
ST-GCN+channel augmentation [103]	-	2020	ST-GCN+new features from parameterized curve	-	91.3	83.4	-	77.3
RHCN+ACSC + STUFE [182]	-	2020	CNN, skeleton alignment	-	92.5	86.9	-	-
[188]	-	2020	Use MS-G3D to extract features, multiple person	-	-	-	-	-
MS-TGN [57]	-	2020	Multi-scale graph	37.3	95.9	89.5	-	-
MM-IGCN [126]	-	2020	Attention, skip connection, TCN	-	96.7	91.3	-	88.8
SlowFast-GCN [104]	-	2020	Two stream with 2 temporal resolution, ST-GCN backbone	-	90.0	83.8	-	-
VE-GCN [63]	-	2020	CRF as loss, distance-based partition, learn edges	-	95.2	90.1	-	84.5
RV-HS-GCNs [187]	-	2020	GCN, interaction representaion	-	96.61	93.79	-	88.2
MS-G3D [55]	Github	2020	Dialted window, GCN+TCN	38	96.2	91.5	86.9	88.4
WST-GCN [105]	-	2020	Multi ST-GCN, ranking loss	-	89.8	79.9	-	-
MS-AAGCN+TEM [66]	-	2020	Extended TCN as TEM	38.6	96.5	91	-	-
ST-PGN [64]	-	2020	GRU, PGNs+LSTM	-	-	-	-	-
GCN-NAS [116]	Github	2020	GRU, PGNs, LSTM	37.1	95.7	89.4	-	-
Poincare-GCN [106]	-	2020	ST-GCN on Poincare space	-	96	89.7	-	80.5
ST-TR [125]	Github	2020	Spatial self-attention + temporal self-attention	-	96.1	89.9	-	81.9
S-STGCN [123]	-	2020	Skip connection, self-attention	-	-	-	-	-
MS-AAGCN [73]	Github	2020	Attention	37.8	96.2	90	-	-
HSR-TSL [62]	-	2020	Skip connection, skip-clip LSTM	-	92.4	84.8	-	-
PA-ResGCN [119]	Github	2020	Part attention	-	96.0	90.9	-	87.3
3s RA-GCN [82]	Github	2020	Occlusion, select joints, ST-GCN backbone	-	93.6	87.3	-	81.1
IE-GCN [107]	-	2020	*L*-hop neighbours, skip connection, ST-GCN backbone	35.0	95.0	89.2	-	-
FV-GNN [195]	-	2020	FV encoding, ST-GCN as feature extractor	31.9	89.8	81.6	-	-
GINs [115]	-	2020	Two skeletons, transfer learning (teacher–student)	-	-	-	-	-
MV-IGNet [59]	Github	2020	Two graphs, multi-scale graph	-	96.1	88.8	-	83.9
GCLS [127]	-	2020	Spatial attention, channel attention	37.5	96.1	89.5	-	-
AMCGC-LSTM [92]	-	2020	LSTM, point, joint and scene level transformation	-	87.6	80.1	-	71.7
GGCN+FSN [91]	-	2020	RL, TCN, feature fusion based on LSTM	36.7	95.7	90.1	-	85.1
ST-GCN-PAM [48]	Github	2020	Pairwise adjacency, ST-GCN backbone interaction	41.68	-	-	76.85	73.87
CGCN [60]	-	2020	ST-GCN backbone	37.5	96.4	90.3	-	-
FGCN [138]	-	2020	Dense connnection of GCN layers	-	96.3	90.2	-	85.4
PGCN-TCA [74]	-	2020	Learn graph connections, spatial+channel attention	-	93.6	88.0	-	-
Dynamic GCN [80]	-	2020	Seperable CNN as CeN to regress adjacency matrix	37.9	96.0	91.5	-	87.3
PeGCN [93]	Github	2020	AGCN backbone	34.8	93.4	85.6	-	-
CA-GCN [196]	-	2020	Directed graph, vertex information aggregation	34.1	91.4	83.5	-	-
SFAGCN [99]	-	2020	Gated TCN	38.3	96.7	91.2	-	87.3
2s-AGCN+PM-STFGCN [109]	-	2020	Attetion, AGCN/ST-GCN backbone	38.1	96.5	91.9	-	-
2s-TL-GCN [86]	-	2020	*d*-distance adjacency matrix, ST-GCN/AGCN backbone	36.2	95.4	89.2	-	-
2s-WPGCN [52]	-	2020	5-parts directed subgraph, GCN backbone	39.1	96.5	91.1	-	87.0
SAGP [124]	-	2020	Attention, spectral sparse graph	36.6	96.9	91.3	-	67.5
JOLO-GCN (2s-AGCN) [54]	-	2020	Descriptor of motion, ST-GCN/AGCN backbone	38.3	98.1	93.8	-	87.6
Sem-GCN [128]	-	2020	Attention, skip connection, *L*-hop, semantics	34.3	94.2	86.2	-	-
SGN [135]	Github	2020	Use semantics (frame + joint index)	-	94.5	89.0	-	79.2
Hyper-GNN [71]	-	2021	Add hyperedges, attention, skip connection	37.1	95.7	89.5	-	-
SEFN [121]	-	2021	Multi-perspective Attention, AGC+TGC block	39.3	96.4	90.7	-	86.2
Sym-GNN [95]	-	2021	Multiple graph, one-hop, GRU	37.2	96.4	90.1	-	-
PR-GCN [183]	Github	2021	MCNN, attention, pose refinement	33.7	91.7	85.2	-	-
GCN-HCRF [90]	-	2021	HCRF	-	95.5	90.0	-	-
2s-ST-GDN [122]	-	2021	GDN, part-wise attention	37.3	95.9	89.7	-	80.8
STV-GCN [198]	-	2021	ST-GCN to obtain emotional state, KNN	-	-	-	-	-
MMDGCN [137]	-	2021	Dense GCN, ST-attention	37.6	96.5	90.8	-	86.8
CC-GCN [131]	-	2021	CNN, generate new graph	36.7	95.33	88.87	-	-
SGCN-CAMM [84]	-	2021	GCN, redundancies, merge nodes by weighted summation of original nodes	37.1	96.2	90.1	-	-
DCGCN [110]	Github	2021	ST-GCN backbone, attentioned graph dropout	-	96.6	90.8	-	86.5
